# RKER-012, a modified ActRIIB-Fc ligand trap with BMP sparing properties, attenuates pathological features of experimental pulmonary arterial hypertension

**DOI:** 10.3389/fcvm.2026.1827438

**Published:** 2026-06-24

**Authors:** R. Keith Babbs, Jeanne Ishimwe, Chris Materna, ffolliott M. Fisher, Tandicka Nurse, Cynthia Pinkus, Pritesh Jain, Kevin Dagbay, Rosa Grenha, Tyler Daman, Claire C. Tseng, Emily A. Ledoux, Evan Lema, Alana Gudelsky, Francis Wolenski, Harveen D. Natarajan, Lorena Lerner, Jennifer L. Lachey, Jasbir Seehra, Sachindra R. Joshi

**Affiliations:** Keros Therapeutics, Lexington, MA, United States

**Keywords:** activin receptor ligand trap (ActRIIB-Fc), bleeding, bone morphogenetic protein (BMP), pulmonary arterial hypertension (PAH), thrombocytopenia and erythrocytosis, vascular integrity

## Abstract

Overactive activin/growth and differentiation factor (GDF) signaling is one of the many pathogenic drivers of progressive vascular remodeling and right ventricular (RV) failure in pulmonary arterial hypertension (PAH). In contrast, bone morphogenic proteins (BMPs) play a crucial role in maintaining vascular homeostasis. The clinical success of sotatercept, an activin receptor (ActR)IIA-Fc ligand trap, has paved the way for targeting activin/GDF signaling in PAH. However, sotatercept has limitations, including severe thrombocytopenia and erythrocytosis, which may increase the risk of hyperviscosity syndrome (HVS) and bleeding. Here, we demonstrated that a modified ActRIIB-Fc ligand trap, KER-012/RKER-012, exhibited BMP-sparing properties while retaining binding to activin/GDF ligands, similar to the wild-type ActRIIA and ActRIIB ligand traps. We also demonstrated that hypoxia increased susceptibility to HVS-related bleeding and mortality in response to erythropoietin (EPO)-induced erythrocytosis and severe thrombocytopenia in Sugen rats. Unlike sotatercept, KER-012/RKER-012 did not elicit erythrocytosis or thrombocytopenia; therefore, a modified ActRIIB-Fc ligand trap like KER-012/RKER-012 could reduce the risk of potential HVS effect and bleeding. We also showed that RKER-012 can attenuate experimental PAH by targeting multiple components of a complex pathobiology, such as dysregulated cell growth, endothelial-to-mesenchymal transition (EndoMT), extracellular matrix (ECM) remodeling, inflammation, immune cell modulation, and fibrosis. This evidence highlights the potential to optimize an activin receptor ligand trap to target overactive activin/GDF signaling in PAH while sparing BMP signaling and minimizing the risk of bleeding, vascular integrity, and erythrocytosis. Ultimately, this approach could improve pulmonary vascular remodeling to alleviate PAH.

## Introduction

Pulmonary arterial hypertension (PAH) is a debilitating disorder characterized by elevated pulmonary artery pressure and right ventricular (RV) dysfunction and failure. The primary pathological feature driving disease progression is pulmonary vascular remodeling, which increases vascular resistance and RV afterload. Key factors contributing to the disease include dysregulated cell proliferation and apoptosis, activation of endothelial-to-mesenchymal transition (EndoMT), remodeling of the extracellular matrix (ECM), inflammation, modulation of immune cells, and fibrosis. These mechanisms collectively lead to the harmful tissue remodeling observed in pulmonary vasculopathy and cardiomyopathy (1–6).

Imbalance of TGF-ß/Activin/GDF-mediated SMAD2/3 and BMP-induced SMAD1/5/9 signaling pathways has been implicated in the pathogenic tissue remodeling, including pulmonary vasculopathy and cardiomyopathy. Overactive SMAD2/3 signaling is one of the many pathogenic contributors in PAH and other cardiovascular abnormalities, including heart failure. Whereas, insufficient signaling through SMAD1/5/9 can occur due to mutations in the BMP type 2 receptor (BMPR2), reduced BMPR2 protein levels, reduced BMP ligand availability, or decreased levels of other signaling molecules associated with SMAD1/5/9 and SMAD4. This deficiency results in an imbalance that promotes overactive signaling through SMAD2/3, resulting in the sequelae of pathological events leading to PAH. Loss-of-function mutations in the BMPR2 are present in over 70% of cases of heritable PAH (7, 8). Additionally, in the absence of these mutations, human and experimental PAH is associated with diminished BMPR2 expression and signaling. Intervention with sotatercept, an ActRIIA-Fc ligand trap, showed that the balance in SMAD2/3 and SMAD1/5/9 signaling can be restored by trapping elevated Activin/GDF ligands and rescuing cardiopulmonary pathologies in preclinical and clinical settings (9–13). Similarly, in animal models of heart failure, targeting overactive SMAD2/3 signaling with other activin receptor pathway inhibitors, such as ActRIIB-Fc, ActRIIB/Alk4-Fc, and an antibody against ActRIIA/ActRIIB, has been shown to be cardioprotective (13–15).

The clinical success of sotatercept in PAH has established a foundation for targeting activin/GDF signaling, transforming the canonical therapeutic approach in PAH and potentially in PH associated with left heart diseases. However, sotatercept has limitations, including severe thrombocytopenia and erythrocytosis, which, if severe, may increase the risk of hyperviscosity syndrome (HVS) and bleeding (16). BMPs are important for maintaining vascular homeostasis (17). Ligand traps with higher affinity to BMPs are known to disrupt vascular integrity, including epistaxis, arteriovenous malformations (AVMs), and hereditary hemorrhagic telangiectasia (HHT)-like phenomenon (18–20). Sotatercept, in addition to binding Activins and GDFs, also binds to BMPs (21). Therefore, we hypothesized that a modified Activin/GDF ligand trap could overcome these potential limitations and attenuate experimental PAH pathology. To test this hypothesis, we engineered an Activin/GDF ligand trap to improve pulmonary vasculopathy by reducing overactive SMAD2/3 signaling while sparing BMP signaling, without inducing erythrocytosis or severe thrombocytopenia. In this study, we describe a novel modified ActRIIB-Fc ligand trap (KER-012) and its murine analog, RKER-012, with potential to improve cardiovascular health in PAH, specifically targeting ligands that contribute to its pathophysiology while minimizing potential interference with the beneficial effects of BMP signaling. Through protein engineering, we developed a modified ActRIIB-Fc ligand trap that minimizes BMP binding, which is critical for maintaining vascular integrity, while retaining binding to Activin/GDF ligands, similar to the wild-type ActRIIA and ActRIIB ligand traps. Furthermore, we evaluated the BMP-sparing properties of RKER-012 and compared it with RAP-011 (murine analog of sotatercept, ActRIIA-Fc) in the inhibition of BMP/ALK1-dependent vascular outgrowth in the neonatal mouse model. We explored KER-012/RKER-012's effect on erythrocytosis in cynomolgus monkeys and in a Sugen hypoxia (SuHx) rat model of PAH, while the therapeutic effects of RKER-012 on pulmonary vasculopathy, RV dysfunction and remodeling were evaluated in a SuHx rat model of PAH. In addition, we evaluated a direct cardioprotective effect on RV in a pulmonary artery banding (PAB)-induced RV failure model. Also, KER-012 was explored in a Phase 1 clinical trial in healthy post-menopausal women. While clinically healthy, this population is at greater risk for subclinical disease, including heart disease, which could potentially provide information on the mechanism of action and proof-of-biology of KER-012 in disease processes. An exploratory serum proteomics assay was conducted in this study to identify biomarkers of KER-012 activity and proteomic changes that align with the translatability of pharmacological properties from rodent models to humans.

## Methods

### Rational protein design and KER-012 *in vitro* characterization

KER-012 is a homodimeric Fc-fusion therapeutic protein consisting of a modified Activin Receptor Type IIB (ActRIIB) extracellular domain (ECD) fused through a short linker peptide to a human immunoglobulin G1 Fc domain (IgG1), including the hinge region, CH2, and CH3 domains. The modification in the ECD was carried out through rational protein design, which involved deletion/substitution of specific amino acid residues (22). This approach was implemented in the wild-type ActRIIB ECD to introduce a higher positive charge at the receptor-ligand interface, which aimed to reduce BMP9 binding. RKER-012 is a research analog of KER-012, which has a murine IgG2a Fc in place of the human IgG1 Fc domain found in KER-012. KER-012 and RKER-012 have the same ECD, which is the domain responsible for ligand binding. Ligand binding affinities were determined for a panel of TGF-ß ligands using surface plasmon resonance (SPR), in comparison to a control, wild-type ActRIIB ECD Fc. In addition, we evaluated ligand-dependent signaling inhibition in a cellular assay (half-maximal inhibitory concentration, IC_50_) to determine the potency of each variant against the same panel of ligands.

### Recombinant protein expression and purification

The modified extracellular domain of ActRIIB was gene-synthesized, PCR amplified and cloned into a mammalian expression vector for production. KER-012 is produced using recombinant DNA technology in Chinese hamster ovary (CHO) cells. Supernatant from conditioned medium is collected and purified through subsequent downstream processing steps, including column chromatography, protein concentration, and tangential flow filtration. The production cell culture is harvested using a pre-sterilized disposable filter, and supernatant is then purified by affinity chromatography with MabSelectSure Protein A resin (Cytiva), followed by a glycine elution. Fractions containing pure, recombinant protein are characterized, pooled, concentrated, and buffer exchanged to 1xTBS using a TFF system (Formulatrix).

### Surface plasmon resonance

The binding kinetics of ActRIIB variants to select ligands were determined by surface plasmon resonance (SPR) using a Biacore T200 system (GE Healthcare, Cytiva). Briefly, the anti-human IgG antibody was diluted in sodium acetate, pH 5.0, and immobilized on a CM4 sensor chip (Cytiva, Cat #29104989) using the amine coupling method. Flow cells 2–4 were used for binding analysis, while flow cell 1 was used to measure non-specific binding to the chip surface. Ligand trap-Fc was captured on the sensor chip until approximately 200 RU. Ligands were diluted in HBS-EP + running buffer at concentrations ranging from 0 nM to 100 nM as a series of dilutions, depending on the ligand being investigated. A ligand was then injected at a flow rate of 40 μL/min for 60 s through all flow cells to measure the ligand binding on-rate, and then running buffer continued to flow through the flow cells for an additional 160–180 s to measure the off-rate. Any binding seen in reference flow was subtracted from the flow cells containing KER-012. The surface was regenerated between each cycle to remove bound protein following the manufacturer's instructions. The kinetic fitting was evaluated using Biacore Insight Evaluation software. The K_on_ (association rate) and K_off_ (dissociation rate) values were used to calculate KD = K_off_/K_on_.

### Cell-based assays

Briefly, assays were performed using the HEK-293-SBE luciferase reporter cells (BPS Bioscience, Cat #60653) to measure SMAD2/3 phosphorylation mediated by each activin and growth differentiation factor type ligand, and C2C12-BRE luciferase reporter cells (23) to measure SMAD1/5/9 phosphorylation mediated by bone morphogenetic protein (BMP) ligands. Cells were plated in a 96-well plate (3 × 10^4^ cells/well) and allowed to adhere overnight. The next day, each ligand trap was titrated in a 96-well plate in DMEM (Gibco, Cat #10567-014) containing 0.1% FBS (Gibco, Cat #16140-071), and the selected ligand was added to each well at a chosen concentration. After diluting KER-012 and the ligand, the dilution plate was sealed and incubated at 37 °C for 1 h. After one hour of incubation, the titration plate was removed from the incubator. Media from the cells was gently aspirated and replaced with 100 µL of preincubated KER-012/ligand mixture. Positive control wells only received ligand, and negative control wells received only starving media. After an additional 21 h in the incubator, the plates were analyzed using the Promega Steady-Glo® Luciferase Assay System according to the protocol provided on a PerkinElmer EnSight Multimode plate reader. After subtracting the average of the negative control wells, inhibition was calculated as the percent of signal loss compared to the averaged positive control wells. Data was plotted in GraphPad Prism and IC_50_ values were calculated using a 4-parameter nonlinear regression fit. Preincubation of cells with appropriate titration of KER-012 provided quantification (via a half-maximal inhibitory concentration [IC_50_] value) of its inhibitory activity against ligand-mediated SMAD signaling for each ligand.

### Animal studies

All animal experiments were approved by the Institutional Animal Care and Use Committee (IACUC) and performed in accordance with the guidelines for the Care and Use of Laboratory Animals published by the U.S. National Institutes of Health. Non-human primate (NHP) studies were conducted at Charles River. The study was reviewed and approved by the facility's IACUC. The facility is accredited by the Association for Assessment and Accreditation of Laboratory Animal Care, International (AAALAC) and registered with the United States Department of Agriculture (USDA).

#### Non-human primate hematological assessment

Briefly, Sysmex XN-100 V was used to measure red blood cell (RBC) count. Hematological assessment was performed in 1 mL of whole blood collected via femoral artery from 2- to 4-year-old cynomolgus monkeys (*n* = 6/sex/group), subcutaneously administered with KER-012 every two weeks at dose levels of 0 (Vehicle), 3, 10, or 50 mg/kg for 190 days.

#### Neovascularization study

Pups from C57BL/6 mice (Inotiv, Indianapolis, IN, USA) were used in this study to evaluate retinal neovascularization. Pups were treated with either vehicle, ALK1-hFc, 10 mg/kg, RAP-011, 10 mg/kg, RAP-011, 20 mg/kg, RKER-012, 10 mg/kg, RKER-012, 20 mg/kg subcutaneously on postnatal day 1 (P1) and P3. On P8 of study, pups were euthanized by hypothermia followed by decapitation according to AVMA guidelines, and retinas were removed, formalin fixed and stained with AlexaFluor® GS-IB4 isolectin (Thermo Fisher, Cat #I21411). Mounted retinas were analyzed by fluorescence microscopy.

#### Erythrocytosis-mediated hyperviscosity study

Sugen-hypoxia rat model was used to evaluate erythrocytosis-mediated hyperviscosity syndrome and its effect on bleeding. Male Sprague Dawley rats (∼250 g, Innotiv, Indianapolis, IN, USA) received a single dose of SU5416 (Sugen, Cayman Chemical, Cat # 13342) (Su, 20 mg/kg sc) and epoetin-alpha (Amgen, Cat # 10038921) (EPO, 7200 U/kg thrice weekly sc) or vehicle under normoxic (SuNx-EPO) or normobaric hypoxic (SuHx-EPO, 10% O₂) conditions (OxyCycler A84XO, Biospherix, Parish, NY, USA) for 5 weeks and compared to normoxia (Nx) controls. Complete blood count, viscosity, bleeding events, and survival were evaluated.

#### Sugen hypoxia rat PAH study

Male Sprague Dawley rats (241–295 g; Charles River) were used in this study for Sugen-Hypoxia model of PAH. Briefly, 200 mg/kg SU5416 (Sugen; Tocris Biosciences; Medkoo Biosciences) suspended in 100% dimethyl sulfoxide (DMSO; JT Baker, Allentown, PA, USA) or Vehicle (DMSO) was administered subcutaneously at study start. Rats were placed in either normoxic (Nx; ∼21% O2) or normobaric hypoxic (Hx; ∼13% O₂) conditions (Hypoxico, New York, NY, USA). Nx rats (*n* = 6) were treated with vehicle, while Hx rats (*n* = 11–12) were treated with either 10 mg/kg RKER-012 or vehicle (TBS) twice weekly sc for 3 weeks. Administration of a similar activin receptor ligand trap at 10 mg/kg twice weekly has previously been shown to effectively block the activin signaling pathway and demonstrate optimal therapeutic efficacy in experimental models of pulmonary hypertension (11–13). In addition, administration of KER-012 at 10 mg/kg dose was previously reported to demonstrate maximum target engagement (24). KER-012 is well tolerated in naïve rats without any adverse effects as high as 50 mg/kg (25), therefore it was not evaluated in Nx rats.

#### Pulmonary artery banding (PAB) study

Male C57BL/6 mice (11–12 weeks old) from the Jackson Laboratory (Bar Harbor, ME, USA) were used in this study for the pulmonary artery banding (PAB) model. Briefly, mouse anesthesia was induced and maintained with isoflurane inhalation (3% and 1.5% in oxygen, respectively). PAB surgery was performed in anesthetized mice by placing a suture ligature around the pulmonary artery as described previously (26). Postoperative analgesia was provided by subcutaneous administration of buprenorphine hydrochloride (0.05–0.1 mg/kg) twice a day for 3–5 days. Sham-operated animals were treated with vehicle (*n* = 15), while PAB mice were treated with either vehicle (*n* = 14) or RKER-012 (*n* = 15; 10 mg/kg; i.p.) twice weekly for 3 weeks.

### Retinal dissection, staining and analysis

Eyes were removed and fixed for 15 min in 4% paraformaldehyde (Thermo Fisher, Cat #J61899). Following fixation, eyes were rinsed in Phosphate Buffer Saline, pH 7.4, and placed on ice. Retinas were dissected, placed in methanol (Fischer Scientific, Cat #A452-4), and stored at −20 °C until staining. Retinas were rinsed with PBS after removing methanol, then incubated for 1 h in a permeabilization and blocking buffer. Permeabilization and blocking buffer (Fischer Scientific, Cat #50-196-4397) were removed and replaced with AlexaFluor® GS-IB4 isolectin (Thermo Fisher, Cat #I21411), and the mixture was incubated overnight at 4 °C. Retinas were rinsed in wash buffer, PBS with 0.3% Triton (Thermo Fisher, Cat #A16046.AP), and whole-mounted using Prolong mountant (Thermo Fisher, Cat #P36930) for analysis. Quantitative analysis was performed on retinal tissue stained with fluorescent isolectin. Images of whole-mounted retinas were obtained using a ZEISS Axioscan 7 at a magnification of 5X. The vascular front ratio was quantified as the ratio of vascular radius (distance from the optic disc to the vascular front) to retinal radius (distance from the optic disc to the edge of the retina). All measurements were performed using ZEISS ZEN 3.8 software.

### Complete blood count analysis and assessment of blood viscosity

Complete blood count (CBC) was examined using the Sysmex XN-1000 hematology analyzer (Sysmex, Lincolnshire, IL) weekly using blood samples collected via tail vein (100–150 µL). Blood viscosity was assessed by measuring its optical density at 700 nm with a spectrophotometer.

### Echocardiography

Cardiac function was assessed by transthoracic echocardiography (GE NextGen Logiqe, 22 MHz linear transducer) in lightly anaesthetized PAB or sham-operated mice, as previously described (27). Briefly, anesthesia was induced and maintained with isoflurane inhalation (3% and 1.5% in oxygen, respectively). M-mode and B-mode echocardiograms were used to measure right ventricle free wall thickness (RVFWT) from the left parasternal long-axis view. Three to five beats were averaged for each mouse. Studies and analyses were performed by investigators blinded to treatments.

### Measurement of systolic pulmonary artery pressure

Rats were anaesthetized by ketamine/xylazine (40–80/10 mg/kg), then an incision was made in the neck, and the jugular vein was isolated and ligated anteriorly. A fluid-filled pressure catheter was introduced into the right jugular vein towards the right ventricle and push forward to the pulmonary artery to measure pulmonary artery pressure (PAP) as described previously (28). Systolic pulmonary artery pressure (sPAP) was monitored using the Notocord HEM data capture system (InStem, USA) for approximately 5–10 min until stable measurements were obtained. At the conclusion of the terminal procedure, exsanguination was performed under deep ketamine/xylazine anesthesia and en-bloc heart and lungs were collected.

Right heart catheterization was performed in mice to confirm elevated pulmonary artery pressure as result of PAB. Briefly, in mice, anesthesia was induced and maintained with isoflurane inhalation (3% and 1.5% in oxygen, respectively) at the end of the experiment. Respiration was supported with a small-animal ventilator. An incision was made at the midline of the upper abdomen, and the diaphragm was opened to expose the heart. A 1.1 French tip solid-state Millar catheter was inserted into the right ventricle from the apex to evaluate systolic pulmonary artery pressure. At the conclusion of the experiment, exsanguination was performed under deep isoflurane anesthesia and en-bloc heart and lungs were collected.

### Measurement of cardiac fibrosis

Hearts were fixed in 10% neutral buffered formalin (NBF), embedded in paraffin, and sectioned at 6 µm, as described previously (29). One middle section per heart was stained with Masson's trichrome. The fibrotic blue area and the entire area were measured using computerized planimetry (Image J, NIH). The fibrotic area was presented as a percentage of the fibrotic area to the entire area. Three random fields per heart were counted and averaged. A total of 30–40 fields per group were measured, and the observer was blinded to the origin of the cardiac sections.

### Lung histopathology

To determine the degree of pulmonary remodeling, lungs were perfused with 10% neutral buffered formalin (NBF), fixed for 48 h, and then transferred to 70% ethanol. Lungs were paraffin-embedded and sectioned at 5 µm. Sections were stained with Hematoxylin and Eosin (H&E) and immunohistochemically stained to detect αSMA (Abcam rabbit polyclonal anti-alpha smooth muscle actin; ab5694, visualized with DAB) with an elastin counterstain. H&E-stained slides were examined for histopathological findings of alveolar inflammation, perivascular/vascular inflammation, vascular necrosis/thrombosis, and smooth muscle hypertrophy of small arterioles and scored on a 0–4 scale. Using the *α*SMA/elastin-stained slides, 100 arterioles between 10 µm- and 50 µm-diameter per animal were categorized as fully muscularized, partially muscularized, or non-muscularized. The pathologist was blinded to the study design during slide evaluation. For 3-plex immunofluorescence (IF) staining of CD31 + SMA + CTGF in formalin fixed paraffin embedded (FFPE) rat lung, staining was conducted on the Leica Bond RX platform. For antigen retrieval, slides were heated in a pH 6 citrate-based buffer for 2 h at 70 °C, followed by a 45-minute incubation with CD31 antibody (1:100, Abcam, Cat #ab182981). CD31 antibody binding was detected using an HRP-conjugated anti-rabbit secondary polymer, followed by opal 690 reagent tyramide signal amplification to allow for immunofluorescence visualization. A second antigen retrieval was done by applying a pH 6 Citrate-based buffer for 25 min at 94 °C to strip the antibody from the first round. The second primary antibody, SMA, was incubated for 30 min (1:2000, Abcam, Cat #ab124964), and antibody binding was detected using an HRP-conjugated anti-rabbit secondary polymer, followed by opal 570 reagent tyramide signal amplification. A third antigen retrieval was performed using a pH 9 EDTA-based buffer for 25 min at 94 °C to strip the antibody from the second round. The third primary antibody, CTGF (1:200, Abcam, Cat #ab227180), was incubated for 30 min, and antibody binding was detected using an HRP-conjugated anti-rabbit secondary polymer, followed by opal 520 reagent tyramide signal amplification. Spectral DAPI (Akoya Biosciences, Cat # FP1490) was used to visualize nuclei. IF slides were imaged using the PhenoImager™ HT automated quantitative pathology imaging system (Akoya Biosciences®) at 500 nm/pixel resolution using a 0.75 NA air objective. Exposure times were optimized for each fluorescent channel to collect emission light within the linear dynamic range of the detection camera. Pulsed LEDs provided excitation for the fluorophore, and a combination of excitation and emission filters eliminated unwanted crosstalk between channels.

### Real-time quantitative PCR

Total RNA was isolated from frozen left lung and right ventricle and homogenized in TRIzol reagent using the Zymo RNA Miniprep kit (Zymo Research, Cat #R2052). RNA concentration and quality was assessed using Nanodrop One (Thermo Scientific, Cat # 13-400-525). Between 250 and 500 ng of total RNA was reverse transcribed to cDNA using the QuantiTect Reverse Transcription Kit (Qiagen, Cat #205313). Quantitative polymerase chain reaction (PCR) was performed with 4 ng cDNA using PowerUp SYBR Green Master Mix (ThermoFisher, Cat #A25741). Relative expression of each gene of interest was determined using the *ΔΔ*CT method and normalized to the relative expression of TBP, 36B4, or B2 m. Sequences of primers are provided in Supplementary Table S1. Samples with insufficient quantity or quality were excluded from analysis.

### Serum analysis from phase 1 study

Serum samples from a Phase 1 trial (ACTRN12621001077853) in healthy post-menopausal women who received either a single dose of KER-012 (4.5 mg/kg) or placebo were evaluated for a comprehensive serum-wide proteomic analysis. Studies involving humans were approved by the Nucleus Network Pty Ltd Alfred Health Human Research Ethics Committee. The Alfred, 55 Commercial Rd., Melbourne, Victoria, Australia, 3004. The studies were conducted in accordance with local legislation and institutional requirements, and participants provided written informed consent.

Serum samples assessed were from individuals who received a placebo (*n* = 5) on day 1 (prior treatment administration, consider baseline) and day 15 and in individuals who received KER-012 at 4.5 mg/kg (*n* = 6) on day 1 (prior treatment administration, consider baseline), day 7, day 15, day 22, and day 29. All samples were measured with the SomaScan® platform (SomaScan® Proteomics) (http://www.somalogic.com) (SomaLogic®, Boulder, CO), containing 7,596 aptamers (6,408 human protein analytes) providing measurements of the relative binding of the serum sample to each of the aptamers in relative fluorescence units (RFU). Calibration and normalization samples were used following the manufacturer's recommended protocol. Data standardization was performed according to the SomaScan® platform data quality-control protocols. To standardize SomaScan assay results, raw SomaScan assay data were first normalized to remove hybridization variation within a run (hybridization normalization), followed by median signal normalization across all samples to remove other assay biases within the run.

### SomaScan data analysis

Forty serum samples were submitted to SomaLogic, LLC, to measure 6,408 proteins by SomaScan multiplex assay. For this analysis, SOMAmer reagents (aptamers) labeled with a photocleavable linker and biotin were immobilized on streptavidin-coated beads. Each serum sample was incubated with reagents, and serum proteins were allowed to bind, forming SOMAmer-target protein complexes. Unbound proteins were washed away, and bound proteins were photocleaved with UV light, leaving only the reagents representing the once-bound proteins. The reagents were then bound to complementary sequences of DNA hybridization probes on a microarray. Probes were then quantified by fluorescence. The result was measured as relative fluorescent units (RFU) and is directly proportional to the amount of target protein in the original serum sample.

The SomaScan assay results were to correct systematic effects introduced during the DNA hybridization step. A control sequence introduced into the assay before hybridization was used to calculate a scaling factor, and each sample was normalized to this factor. Additionally, median normalization allows comparison of signals across a plate by correcting for assay- or sample-induced variation in total protein concentration. Finally, the median signal intensity from each subarray was used to calculate a sample-based scaling factor.

### SomaScan statistical analysis

Quality control assessment and exploratory data analysis were performed for all samples in this study. Hybridization control normalization, intraplate median signal normalization, median signal normalization to a human serum reference, and plate scaling and calibration were performed by SomaLogic for this data. All samples passed the SomaLogic initial quality control assessments for normalization and calibration (data not shown).

The R package “readat” (Cotton et al., 2016, BMC Bioinformatics) was used to read the SomaScan intensity data. Quality control assessment of proteome profiles was performed using four automated outlier tests and manual inspection of MA and density plots. No samples failed other automated outlier tests.

Baseline normalization was performed by combining placebo day 1 and day 15 samples, and KER-012 day 1 samples, to generate a baseline group. We then compared this baseline group to KER-012 day 7, day 15, day 22, and day 29 combined. Models were fit in R using the limma package, and logFC values were extracted for the placebo versus drug comparison.

### Statistical analysis

Statistical analyses were conducted using GraphPad Prism software (Prism v10, GraphPad Software Inc., San Diego, CA, USA). Statistical differences between experimental groups were evaluated using independent sample unpaired Student's t-tests or One-way ANOVA followed by *post-hoc* analysis (for comparison of more than two groups). *P* < 0.05 was considered significant for all statistical analyses. Statistical outliers were removed using Grubbs’ test.

## Results

### KER-012 showed select TGF-β ligand binding and inhibition

**Figure 1:**
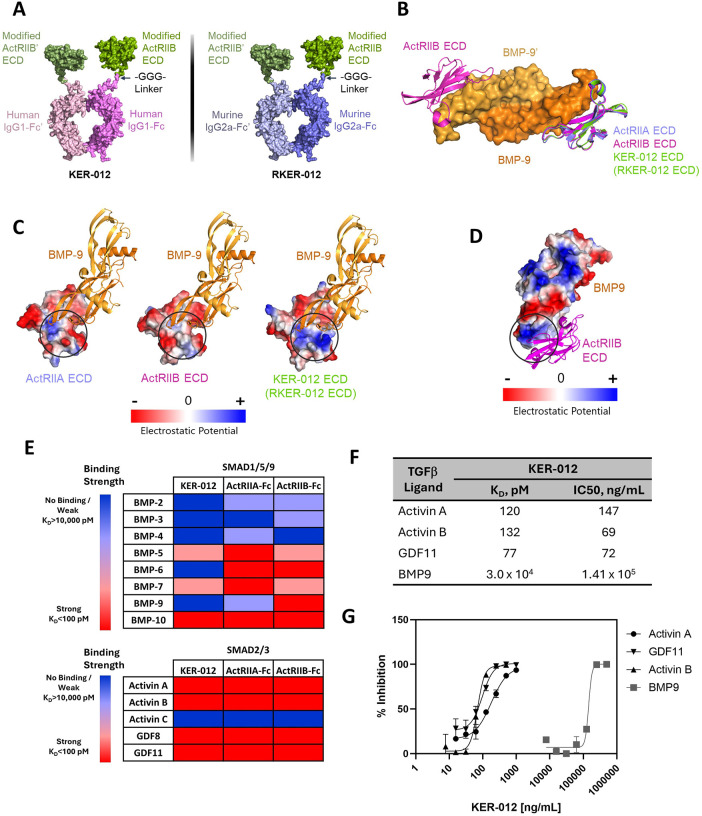
KER-012 showed differential TGF-β ligand binding and cell-based function. **(A)** Schematic of the KER-012 and RKER-012 ligand trap, illustrating the modified ActRIIB extracellular domain (ECD) in green and the antibody constant region in purple for KER-012 and blue for RKER-012. **(B)** The x-ray structure of ActRIIB ECD bound to BMP9 (PDB ID: 4FAO (31)), aligned with the crystal structure of ActRIIA ECD (PDB ID: 7U5P (32)) and the AlphaFold-generated model (30) of KER-012/RKER-012 ECD. **(C)** Electrostatic potential maps of ActRIIA, ActRIIB, and KER-012/RKER-012 ECDs generated using PyMoL, highlighting differences (black circle) in the electrostatic potential distribution at the receptor binding interface. **(D)** Electrostatic potential map of BMP9 generated using PyMOL, highlighting the region within the receptor-ligand interface. In the electrostatic potential map, red indicates negative potential and blue indicates positive potential. White regions correspond to neutral potentials. **(E)** Binding selectivity of KER-012 to relevant TGF-β ligands, comparing SMAD 1/5/9 and SMAD 2/3 signaling with wild-type ActRIIA ECD and ActRIIB ECD. **(F)** The SPR-derived binding affinities and cell-based IC_50_ values of KER-012 for selected TGF-β ligands. **(G)** Representative cell-based inhibition curve showing the percent inhibition of signaling as a function of KER-012 concentration in the presence of selected TGF-β ligands.

KER-012 consists of a modified ActRIIB extracellular domain (ECD) fused to the constant region of human IgG1 via a (Gly)₃ linker. RKER-012 has a murine IgG2a Fc in place of the human IgG1 Fc domain found in KER-012. KER-012 and RKER-012 have the same ECD, which is the domain responsible for ligand binding (Figure 1A). RKER-012 is a research analog of KER-012, used in rodent nonclinical studies to minimize the development of anti-drug antibody responses. In Figure 1A, KER-012 and RKER-012 are modeled using AlphaFold (30) and are shown as a surface representation. To explore the structural basis of the unique binding and potency of KER-012, structural modeling was performed. Alignment of the AlphaFold-predicted model of KER-012/RKER-012 ECD, which shares 91.3% sequence identity with wild-type ActRIIB ECD, revealed a similar overall fold compared to known crystal structures of ActRIIB ECD (PDB: 4FAO) (31) and ActRIIA ECD (PDB: 7U5P) (32), with root-mean-square deviations (RMSD) of 0.389 and 0.559, respectively (Figure 1B). Electrostatic potential maps of the ECDs of ActRII(A/B) receptors and KER-012/RKER-012, generated using PyMOL (33) (Figure 1C), showed that KER-012/RKER-012 has a relatively high positive charge in the receptor-ligand interface, a feature not seen in ActRIIA or ActRIIB. BMP9 also displays a high positive charge in the region that interacts with the receptor (Figure 1D), suggesting that electrostatic repulsion may promote reduction in BMP9 binding to KER-012/RKER-012.

Initial characterization of KER-012 with activin A using negative stain electron microscopy resolved low-resolution structural features and binding orientation (Supplementary Figure 1B). These data suggest that a single KER-012 molecule binds to one homodimer of activin A. The binding affinity profile of KER-012 was compared to wild-type ActRIIA- and ActRIIB-based ligand traps across both off-target (BMPs) and on-target (activins and GDFs) ligands (Figure 1E). The heat map of the binding affinity revealed distinct binding signatures for each ligand trap. KER-012 showed minimal binding to BMP-(2, 3, 4, 6, 9), while exhibiting moderate to strong binding to BMP-(5, 7, 10). The ActRIIA-based ligand trap displayed weaker binding to BMP-(2, 3, 4, 9) but strongly preferred BMP-(5, 6, 7, 10). Notably, KER-012 was the most BMP-9 sparing compared to ActRIIA and ActRIIB. However, all ligand traps tested demonstrated similar strong binding to on-target activins (A, B) and GDFs (8, 11), with weak binding to activin C. The SPR binding characterization confirmed that KER-012 retained binding to activin-(A, B) and GDF-11 with picomolar affinity (Figure 1F), similar to the wild-type ActRIIA and ActRIIB ligand traps (Figure 1E), while showing weaker binding to BMP9 (Figure 1F). In luciferase reporter-based cellular assays for SMAD2/3 and SMAD 1/5/9 signaling, KER-012 potently inhibited SMAD2/3 signaling induced by activin-(A, B) and GDF-11 but was significantly less potent in inhibiting BMP9-induced SMAD 1/5/9 signaling—by approximately 785- to 2000-fold compared to other TGF-β ligands tested (Figure 1F). A representative inhibition curve (Figure 1G) further illustrates the relative potency of KER-012 against selected TGF-β ligands. Taken together, these data indicate that KER-012 possesses a distinct binding and potency profile across TGF-β ligands, differing significantly from those of the wild-type ActRIIA- and ActRIIB-based ligand traps.

Furthermore, sequence alignment of activins, GDFs, and BMP ligands revealed notable differences in the amino acid residues at the receptor-ligand binding interface, as predicted by the known ActRIIB:BMP9 structure (Supplementary Figure S2). These inherent variations in the TGF-β ligand sequences within the receptor:ligand binding interface may account for the differential binding affinities of ligands to both wild-type and modified ActRII-based ligand traps.

### RKER-012 exerts BMP-sparing properties *in vivo*

**Figure 2:**
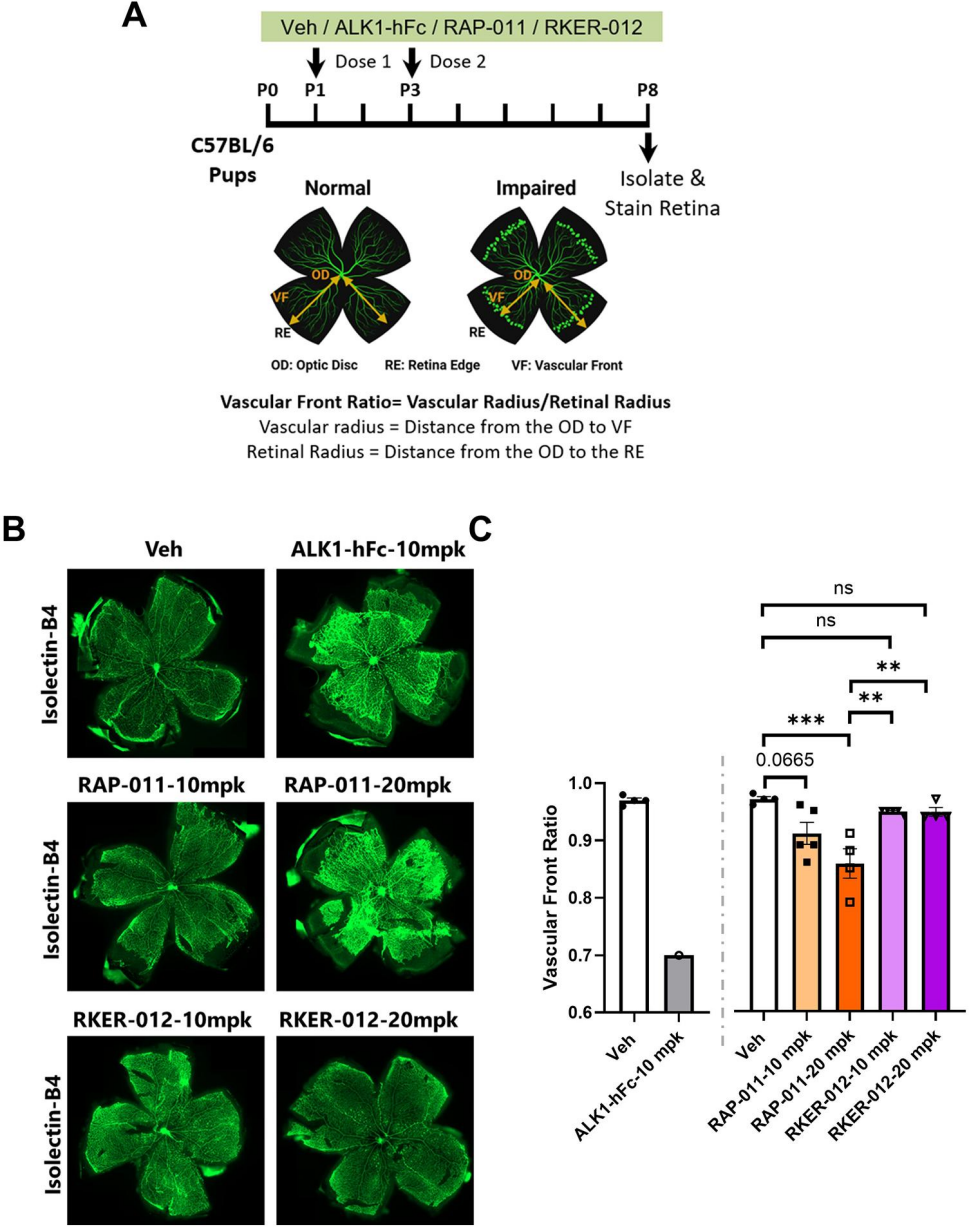
RKER-012 demonstrated BMP sparing properties *in vivo*. **(A)** Experimental approach to evaluate the effect of RKER-012 on retinal neovascularization in C57BL/6 pups. Pups were treated with either vehicle (*n* = 4), ALK1-hFc (10 mg/kg, *n* = 9), RAP-011 (10 mg/kg, *n* = 5 and 20 mg/kg, *n* = 4), and RKER-012 (10 mg/kg, *n* = 5 and 20 mg/kg, *n* = 4) subcutaneously on postnatal days 1 (P1) and 3 (P3). Pups were sacrificed on postnatal day 8 (P8), and isolated retinas were dissected and stained with Isolectin-B4 to visualize the vasculature and measure the vascular plexus. ALK1-hFc-treated pups exhibited a 55% mortality rate. The retinas from the surviving pups were extremely fragile and exhibited increased stiffness, leading to sample loss due to technical challenges. Only one retina passed the quality control for subsequent staining and analysis. Vascular front ratios (the ratio of the vascular radius to the retinal radius) were compared between the vehicle control group and all other groups. **(B)** Representative retina images of neonatal mice treated with ALK1-hFc (10 mg/kg), RAP-011 (10 mg/kg and 20 mg/kg), and RKER-012 10 mg/kg and 20 mg/kg) mpk, mg per kg (mg/kg). **(C)** Quantification of vascular front ratio. Data are presented as mean ± SEM. Analysis was performed using one-way ANOVA followed by Tukey's multiple comparison test: ns: not significant; **p ≤ 0.01; ***p ≤ 0.001.

Previous reports from neonatal mouse models of retinal angiogenesis have elegantly demonstrated that BMP/ALK1 signaling is critical to the development of retinal vasculature (34, 35). Using this model as illustrated in Figure 2A, we evaluated BMP sparing properties of RKER-012, a research analog of cibotercept (KER-012) and RAP-011, a research analog of sotatercept. Activin receptor-like kinase 1 (ALK1-hFc), a BMP9 and BMP10 ligand trap (36, 37), was included as a positive control for vascular disruptions. The vascular front ratio was quantified as shown in Figure 2A to assess the effect of BMP/ALK1-mediated inhibition of vascular development. Retinas from pups treated with ALK1-hFc showed disordered networks of multilayered blood vessels and a reduction in the vascular front ratio (Figures 2B,C). This is indicative of disrupted retinal vascular development (DRVD) and consistent as previously reported (35). RAP-011, the ActRIIA ligand trap, resulted in a dose-dependent reduction in the vascular front ratio, potentially indicative of blunted vascular development. At the 20 mg/kg dose, RAP-011 treated pups had significantly lower (*p* ≤ 0.001) vascular front ratios than those of the vehicle control (Figures 2B,C). In contrast, RKER-012 treated pups at both 10 and 20 mg/kg doses had significantly larger vascular front ratios than the positive control ALK1-hFc and RAP-011 treated mice (Figures 2B,C). In fact, the RKER-012-treated group vascular front ratios were not different from the vehicle controls. The lack of DRVD in mice treated with RKER-012 at both 10 mg/kg and 20 mg/kg suggests that RKER-012 did not inhibit BMP/ALK1-dependent vascular outgrowth, which is consistent with *in vitro* BMP-sparing properties of RKER-012 as shown in Figures 1E–G.

### Erythrocytosis-mediated hyperviscosity syndrome exacerbates bleeding in a sugen-hypoxia rat model

**Figure 3:**
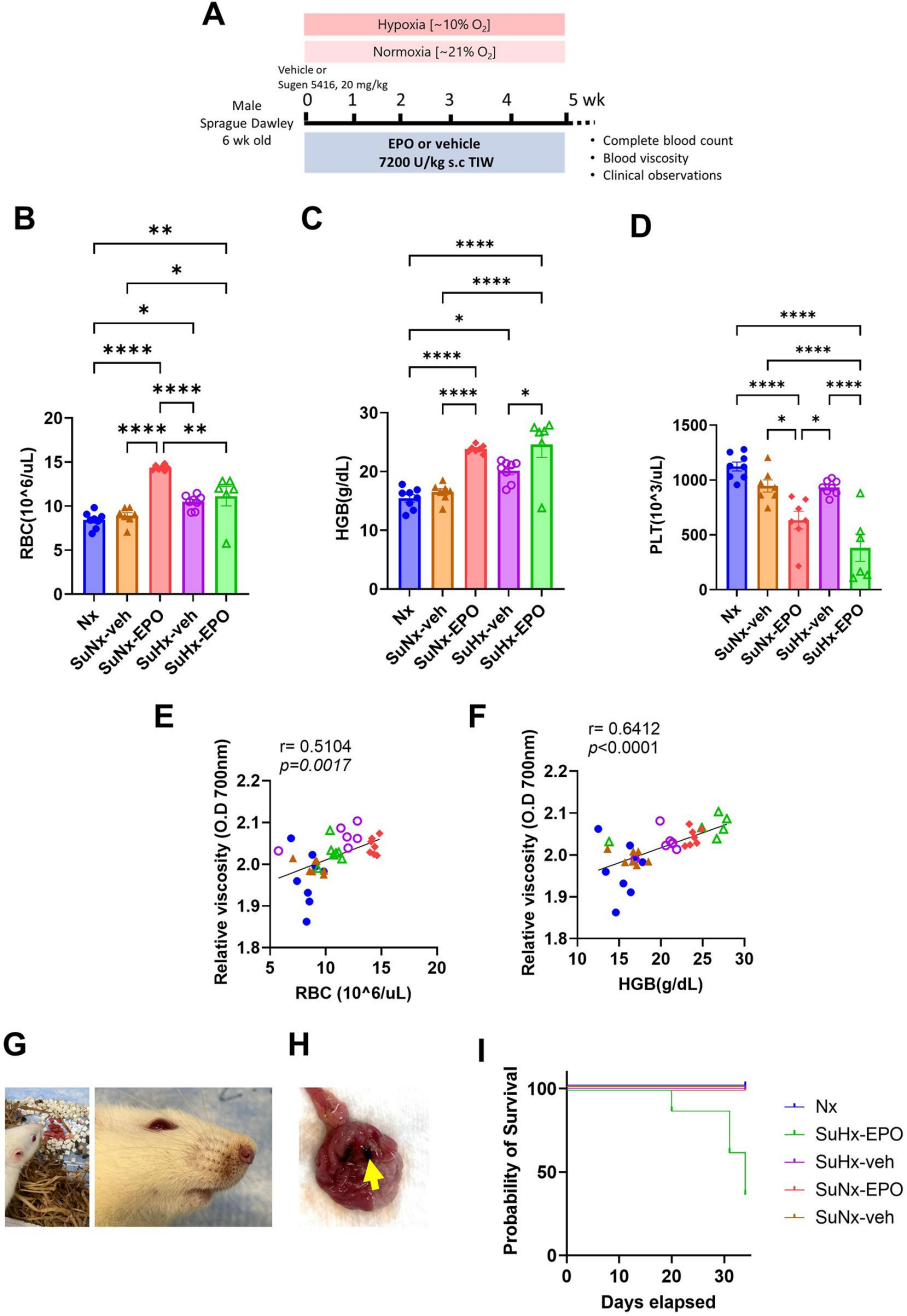
Erythrocytosis-mediated hyperviscosity syndrome exacerbated bleeding in a sugen-hypoxia rat model. **(A)** Experimental approach to evaluate erythrocytosis-mediated hyperviscosity combined with thrombocytopenia exacerbates bleeding in the Sugen-hypoxia rat model. Blood levels of **(B)** red blood cells **(**RBCs), **(C)** hemoglobin (HGB), and **(D)** platelets (PLT). Data are presented as mean ± SEM. Analysis was performed using one-way ANOVA followed by Sidak's multiple comparisons test. *P* values are represented as *p < 0.05, **p < 0.01, **** *p* < 0.0001. Hx: hypoxia, TIW: thrice weekly. **(E)** Positive correlation between RBC number and relative blood viscosity. **(F)** Positive correlation between HGB and relative blood viscosity. Statistical analysis was performed using Pearson correlation test. Pathological observation of **(G)** in-life nosebleed and **(H)** gastrointestinal bleeding observed at terminal necropsy in rats that received Sugen5416 and treated with EPO in hypoxia. **(I)** Kaplan–Meier Survival curve showed that rats in the SuHx-EPO group had significantly reduced survival compared to the Nx. No mortality was observed in the other groups.

KER-012/RKER-012 did not elicit erythrocytosis in non-human primates (Supplementary Figure S3A) and SuHx rats (Supplementary Figure S3B). Sotatercept, on the other hand, elicits erythrocytosis (38–40). To determine whether increased RBC volume and the development of hyperviscosity syndrome could potentiate bleeding risk, as noted in the sotatercept label (16), a SuHx rat model was evaluated for EPO-induced erythrocytosis-mediated hyperviscosity and bleeding risks, as illustrated in Figure 3A. As shown in Figures 3B–D, EPO treatment increased RBCs [SuNx-EPO: +41%, *p* < 0.0001; SuHx-EPO:+24%, *p* < 0.01] and hemoglobin [SuNx-EPO:+35%, *p* < 0.0001; SuHx-EPO:+37%, *p* < 0.0001], and decreased platelets [SuNx-EPO:-77%, *p* < 0.0001; SuHx-EPO:-194%, *p* < 0.0001], compared to non-EPO-treated Nx controls. Increased erythrocytosis [r = 0.6604, *p* < 0.0001] and hemoglobin [r = 0.6813, *p* < 0.0001] positively correlated with increased blood viscosity (Figures 3E,F, respectively). Figures 3G,H show gastrointestinal bleeding and nosebleeds in SuHx-EPO rats. Although SuNx-EPO rats had increased RBCs, bleeding events were not observed. Additionally, thrombocytopenia was more severe with SuHx-EPO compared to SuNx-EPO. Concurrently, elevated RBCs, increased blood viscosity, severe thrombocytopenia, and bleeding events were observed in SuHx-EPO rats, which displayed a reduced survival rate [−62.5%, *p* < 0.0001] (Figure 3I). These data demonstrate that hypoxia increased susceptibility to HVS-related bleeding and mortality in response to EPO-induced erythrocytosis and severe thrombocytopenia in Sugen rats. Altogether, our results showed that increased blood viscosity, partly due to increased erythrocytosis, along with accompanying severe thrombocytopenia, may potentially raise the risk of bleeding in patients with chronic diseases associated with systemic hypoxia, such as PAH. Thus, a modified ActRIIB-Fc ligand trap, such as KER-012/RKER-012, that does not elicit erythrocytosis could reduce the risk of hyperviscosity syndrome.

### RKER-012 exerts protective effects on cardiopulmonary parameters in a sugen-hypoxia rat model of PAH

**Figure 4:**
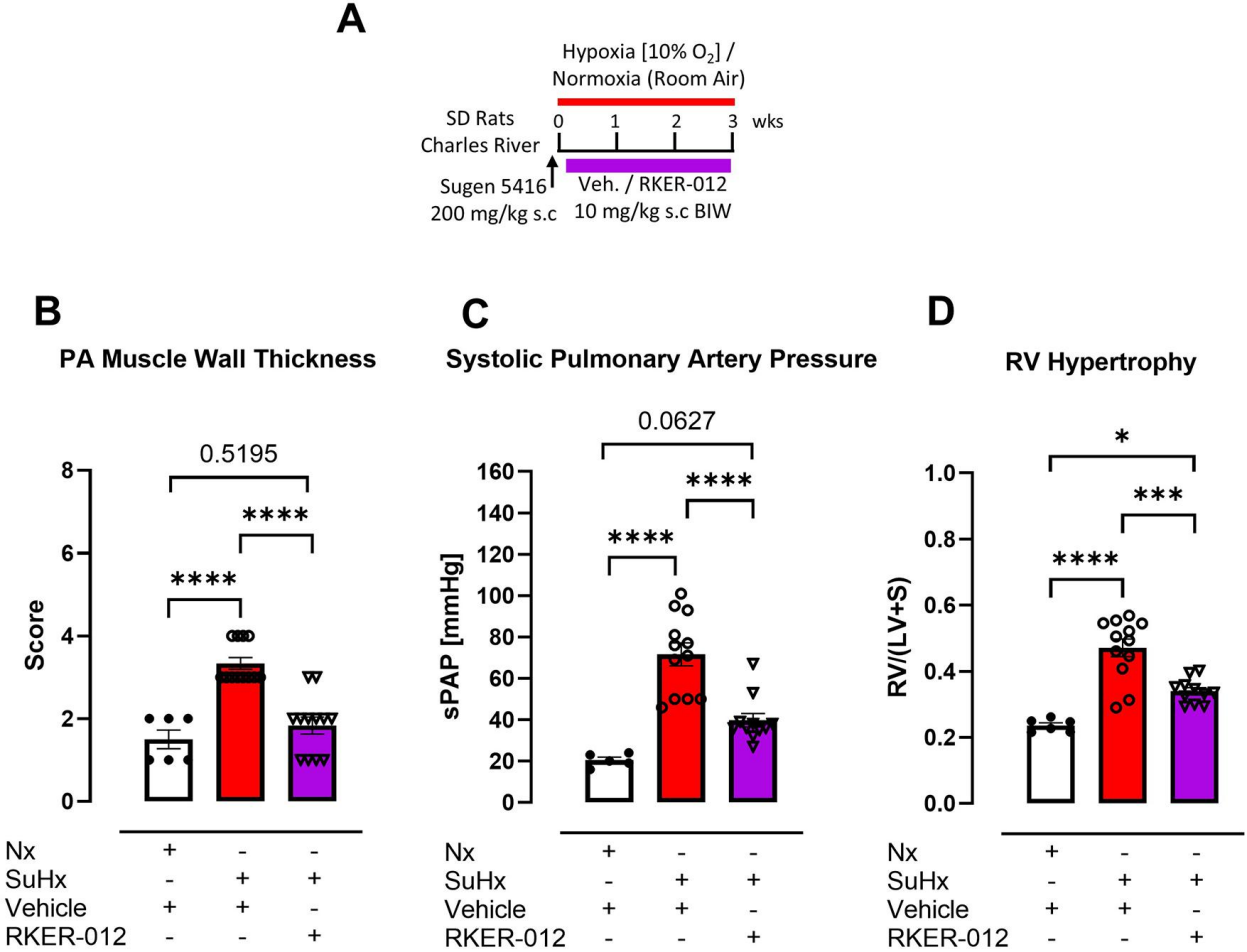
RKER-012 attenuates cardiac hemodynamic parameters in a rat sugen hypoxia rat PAH model. **(A)** Experimental approach to evaluate effects of RKER-012 in attenuating PAH in a SuHx rat model. Rats were treated on day 0 with a single dose of SU5416 (200 mg/kg, s.c.) and exposed to 3 weeks of hypoxia (10% O_2_). Rats receiving SU5416 and exposed to hypoxia were treated twice weekly with RKER-012 (10 mg/kg, s.c.) or vehicle (TBS) for 3 weeks. **(B)** Pulmonary Artery (PA) Muscle Wall Thickness Score, **(C)** Systolic Pulmonary Artery Pressure (sPAP), and **(D)** Right ventricular (RV) hypertrophy as assessed by Fulton Index, ratio of weight of Right Ventricle (RV) to weight of Left Ventricle (LV) and Septum (S) in normoxia rat treated with vehicle (Nx-Veh), SuHx treated with vehicle (SuHx-Veh) or RKER-012 (SuHx-RKER-012). *n* = 5-12 in each group. Data are presented as Mean ± SEM. Analysis was performed using one-way ANOVA and Tukey *post hoc* test (*p < 0.05, ***p < 0.001, ****p < 0.0001).

Reports from SuHx rat model of PAH have shown that RAP-011, an ActRIIA-Fc research analog of sotatercept, exerts protective effects on cardiopulmonary parameters (11, 12). Here, we evaluated the effect of RKER-012, a modified ActRIIB-Fc ligand trap, in SuHx rat model of PAH. We investigated whether RKER-012 can inhibit the development of elevated systolic pulmonary arterial pressure (sPAP) and right ventricular (RV) hypertrophy. Male Sprague Dawley rats were subcutaneously injected with a single dose of Sugen 5416 and exposed to 10% hypoxia for 3 weeks, during which they received either vehicle (SuHx-Veh) or 10 mg/kg RKER-012 biweekly (SuHx-RKER-012) as illustrated in Figure 4A. Control rats were kept in normoxia (∼21% O2) for 3 weeks and treated with vehicle (Nx-Veh). Following 3 weeks of treatment with vehicle or RKER-012, rats were assessed for cardiopulmonary phenotypes. SuHx-Veh rats had significantly increased pulmonary artery muscle wall thickness (Figure 4B) [*p* ≤ 0.0001], systolic pulmonary arterial pressure (sPAP) [+252.2%; *p* ≤ 0.0001] (Figure 4C), and RV hypertrophy [+99.4%; *p* ≤ 0.0001] (Figure 4D), compared to Nx-Veh rats. SuHx rats treated with RKER-012 had significantly decreased pulmonary artery muscle wall thickness [*p* ≤ 0.0001] (Figure 4B), sPAP [−37.4%; *p* ≤ 0.0001] (Figure 4C), and RV hypertrophy [−24.8%; *p* = 0.0001] (Figure 4D) compared to SuHx-Veh. Furthermore, unlike vasodilators, the normalization of pulmonary arterial muscle wall thickness in SuHX-RKER-012 rats to that of Nx-Veh rats (Figure 4B), reinforces the tissue remodeling effect of activin receptor ligand trap, indicative of rebalancing pro-proliferative (Smad2/3) and anti-proliferative (Smad1/5/9) pathway as described previously (7, 11). Because activin receptor ligand traps are not a potent vasodilator, the lack of normalization in sPAP in SuHx-RKER-012 rats (Figure 4C) could potentially be attributed to residual vascular tone that may have persisted. The resultant residual sPAP could potentially be playing a role in the lack of normalization of RV hypertrophy in SuHx-RKER-012 rats (Figure 4D). Nonetheless, these data show that RKER-012 protected against the development of increased pulmonary artery muscle wall thickness, sPAP and RV hypertrophy in a SuHx rat model of PAH.

### RKER-012 attenuates the development of vascular muscularization in SuHx model

**Figure 5:**
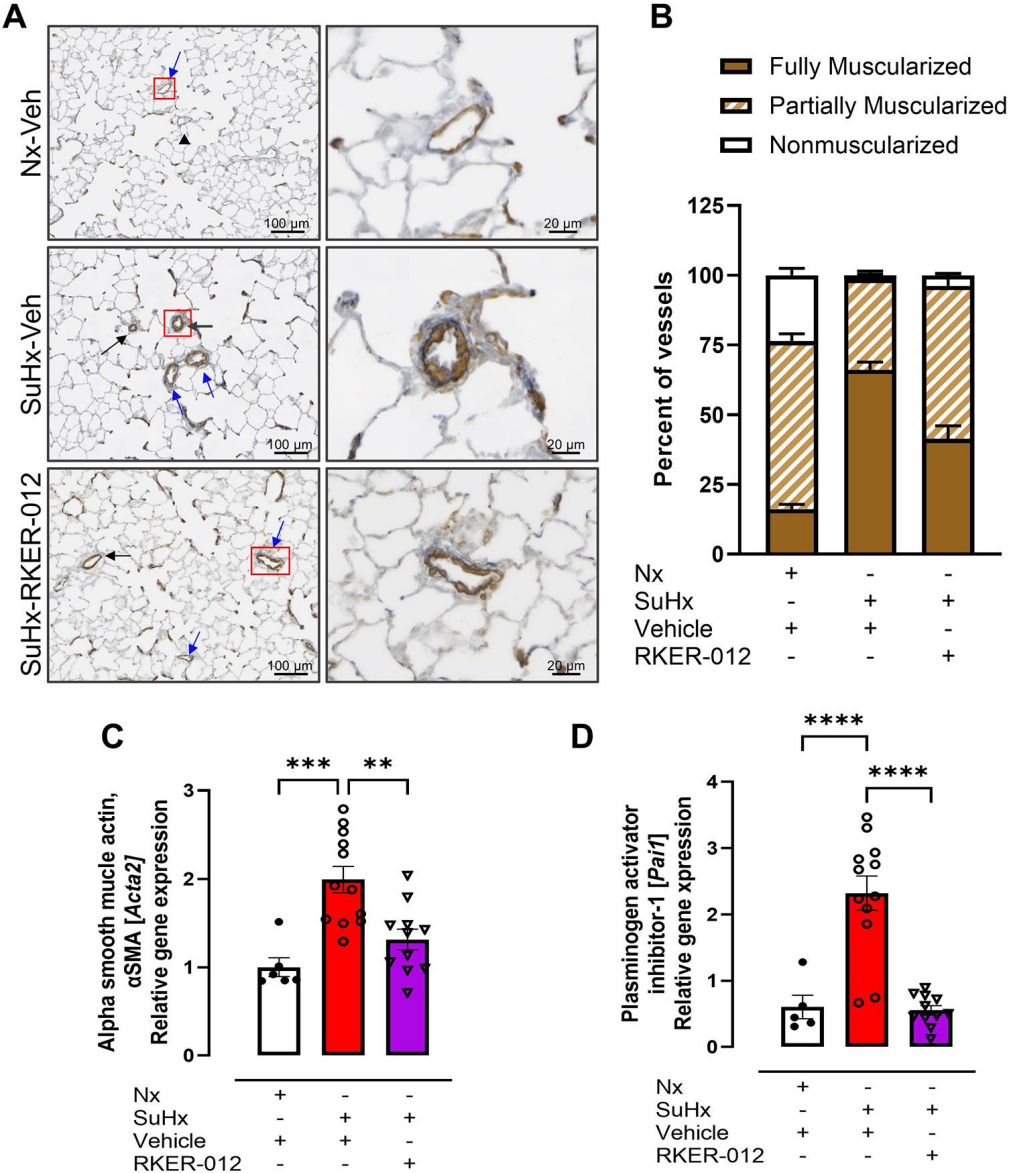
RKER-012 attenuates pulmonary vascular remodeling in an experimental sugen hypoxia rat PAH model. **(A)** Representative images of lung sections stained with αSMA/elastin in normoxic rats treated with vehicle (Nx-Veh), Sugen hypoxia rats treated with vehicle (SuHx-Veh) and Sugen hypoxia rats treated with RKER-012 (SuHx-RKER-012). Arrows in the left images indicates pulmonary vessels, and red box represents the magnified pulmonary vessels in the right image. **(B)** Pulmonary vessel muscularization, **(C)** gene expression levels of alpha smooth muscle actin (αSMA) *Acta2* and **(D)** plasminogen activator inhibitor-1, *Pai-1* in lungs of Nx-Veh, SuHx-Veh, and SuHx-RKER-012. *n* = 6-12 in each group. Data are presented as Mean ± SEM. Analysis was performed by using one-way ANOVA and Tukey *post hoc* test (**p < 0.01, ***p < 0.001, ****p < 0.0001).

Muscularization of non-muscular arteries is one of the key pathological features of PAH. Therefore, we evaluated the vasculo-protective effects of RKER-012 in the muscularization of non-muscular pulmonary arteries in SuHx rat model. In comparison to normoxic controls (Nx-Veh), SuHx-Veh rats showed significantly more muscularized [partial, *p* = 0.0002, full *p* ≤ 0.0001] pulmonary arteries and significantly fewer non-muscularized [*p* ≤ 0.0001] pulmonary arteries (Figure 5B). In contrast, SuHx rats treated with RKER-012 had significantly less muscularized [partial, *p* = 0.0002 and full, *p* ≤ 0.0001] pulmonary arteries compared to SuHx-Veh rats (Figure 5B). Although not statistically significant, non-muscularized [*p* = 0.1215] arteries in the SuHx rats treated with RKER-012 showed an increasing trend. (Figure 5B). These differences can be observed in the representative images shown in Figure 5A. Furthermore, pulmonary expression of the smooth muscle cell marker αSMA, Acta2 was significantly increased in SuHx-Veh [*p* = 0.0003] compared to Nx-Veh. In contrast, SuHx rats treated with RKER-012 showed reduced expression of αSMA, Acta2 [*p* = 0.0022] in comparison to SuHx-Veh rats (Figure 5C). Additionally, plasminogen activator inhibitor-1 Pai1, a marker of vascular remodeling, was increased in SuHx-Veh rats [*p* ≤ 0.0001]. Importantly, SuHx rats treated with RKER-012 showed reduced expression of Pai1 [*p* ≤ 0.0001] in comparison to SuHx-Veh rats (Figure 5D). These results demonstrate that RKER-012 attenuated the development of vascular muscularization in a SuHx rat model of PAH.

### RKER-012 attenuates changes in markers of endothelial dysfunction and prevents EndoMT

**Figure 6:**
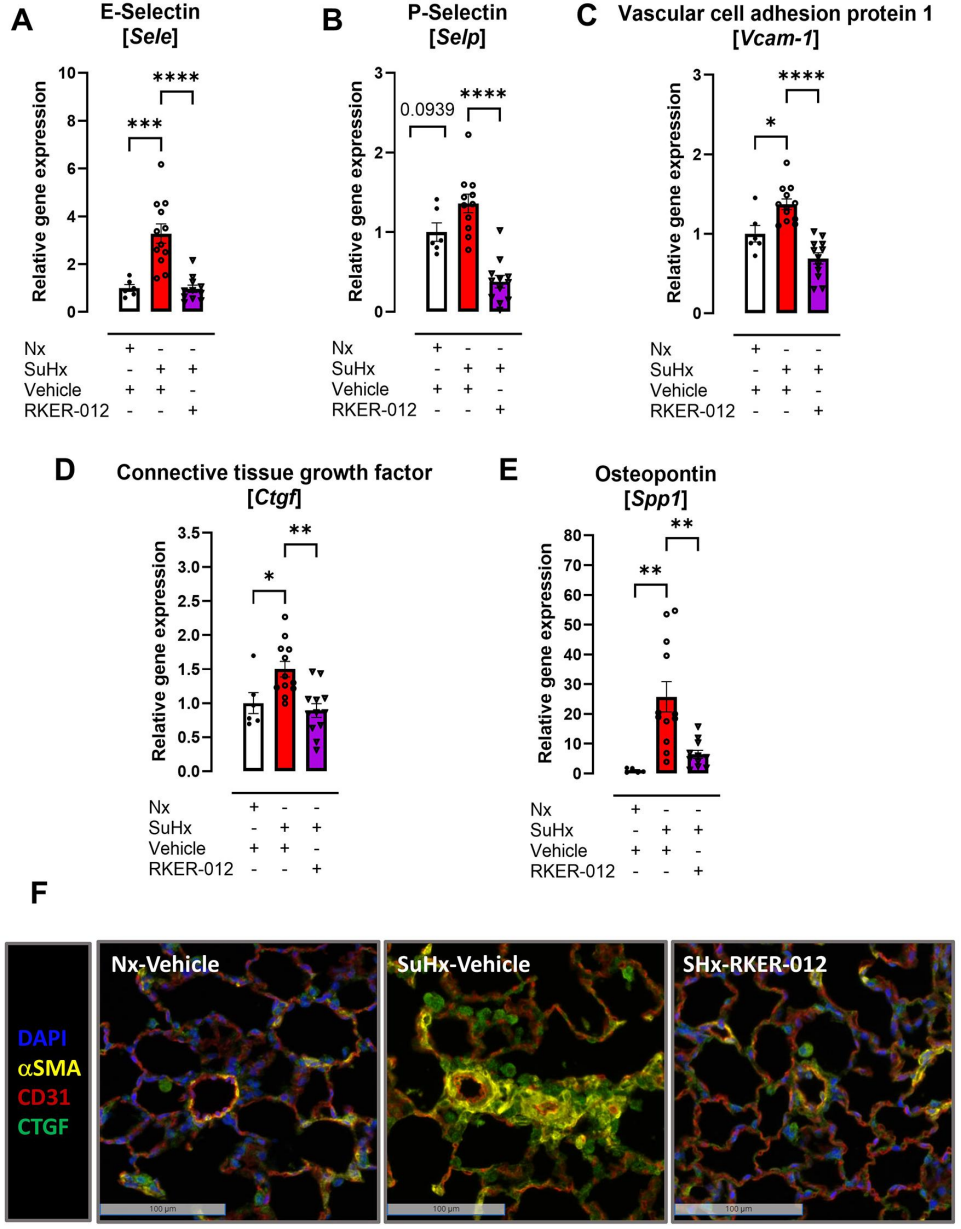
RKER-012 attenuates changes in markers of endothelial dysfunction and prevents endothelial-to-mesenchymal transition. Gene expression of markers associated with EndoMT, including **(A)** E-selectin [*Sele*]*,* **(B)** P-selectin *[Selp],* **(C)** vascular cell adhesion molecule 1 [*Vcam-1*], **(D)** connective tissue growth factor [*Ctgf*], and **(E)** osteopontin [*Spp1*]. Compared with Nx-Veh rats, SuHx-Veh rats showed increased expression of these markers. Treatment with RKER-012 reduced the gene expression of these markers in SuHx-RKER-012 rats. Data are presented as Mean ± SEM. Analysis was performed by using one-way ANOVA and Tukey *post hoc* test (*p < 0.05, **p < 0.01, ***p < 0.001, ****p < 0.0001). **(F)** Representative image of immunohistochemistry of a rat lung section stained with αSMA (Yellow), CD31 (Red), CTGF (Green) and DAPI (Blue), showing normal pulmonary artery (Nx-Veh), remodeled pulmonary artery (SuHx-Veh) and rescued pulmonary artery (SuHx-RKER-012).

Endothelial dysfunction and EndoMT are key features of pulmonary vascular remodeling in PAH. Endothelial cell surface is comprised of various cell adhesion molecules, including E-selectin, P-selectin and vascular cell adhesion molecule-1 (Vcam-1), which are known to facilitate interaction with leukocytes under inflammatory conditions to promote endothelial barrier dysfunction leading to further vascular remodeling and disease progression (41). Levels of E-selectin and P-selectin are elevated in PAH patients (42, 43) and are indicative of endothelial activation and dysfunction (44). Therefore, we evaluated the effect of RKER-012 on gene expression of markers of endothelial dysfunction and EndoMT in the lungs of SuHx rats (Figure 6). Spp1, secreted phosphoprotein 1, also known as osteopontin, modulates various cellular processes, including cell proliferation, migration, apoptosis, extracellular matrix synthesis, and inflammation through interaction with integrins and CD44, a ubiquitously expressed cell-surface receptor, to induce EndoMT (45). Circulating levels of Spp1 have been reported to be elevated in various forms of PH (45). An increase in pulmonary expression of Spp1in PAH patients has been strongly correlated with disease severity (46). Similarly, connective tissue growth factor (Ctgf), a matricellular protein, plays an essential role in tissue remodeling and fibrosis, including activation of myofibroblasts originating from EndoMT (47). In this study, relative to Nx-Veh, SuHx-Veh had significantly increased expression of E-selectin, Sele [*p* = 0.0003] (Figure 6A), vascular cell adhesion molecule-, Vcam-1 [*p* = 0.0102] (Figure 6C), connective tissue growth factor, Ctgf [*p* < 0.05] (Figure 6D), and osteopontin, Spp1 [*p* = 0.0025] (Figure 6E), with a strong trend for elevated P-selectin, Slep [*p* = 0.0939] (Figure 6B). Importantly, lungs of SuHx rats treated with RKER-012 (SuHx-RKER-012) showed reduced expression of E-selectin [*p* < 0.0001] (Figure 6A), P-selectin [*p* < 0.0001] (Figure 6B), Vcam-1 [*p* < 0.0001] (Figure 6C), Ctgf [*p* = 0.0032] (Figure 6D), and Spp1 [*p* = 0.0038] (Figure 6E) in comparison to SuHx-Veh rats. The contrasting observation of lower P-selectin gene expression in SuHX rats treated with RKER-012 than in Nx-Veh rats, unlike that observed with E-selectin, could be attributed to distinct regulatory mechanisms between E-selectin and P-selectin. E-selectin is synthesized *de novo*, while P-selectin is stored in Weibel Palade bodies and rapidly mobilized to the surface, within minutes, upon stimulation, without requiring new protein synthesis (48, 49). In summary, the reduction in the expression of these genes by treatment with RKER-012 suggests that RKER-012 was protective against endothelial dysfunction and EndoMT.

Alpha smooth muscle actin (*α*SMA) is the marker of smooth muscle cells and myofibroblasts, which contribute to pulmonary vascular remodeling by proliferation of cells expressing these markers and narrowing of the vascular lumen (50, 51). During EndoMT, endothelial cells acquire characteristics of mesenchymal cells, including the expression of αSMA (52, 53). Figure 6F showed distinct upregulation αSMA- a marker of smooth muscle cells and myofibroblasts in the remodeled vessels of SuHx-Veh-treated rats compared to the Nx-Veh cohort, demonstrating an increase in smooth muscle cell population, including myofibroblasts, potentially due to EndoMT. Figure 6F also showed distinct upregulation of CTGF in the remodeled vessels of SuHx-Veh-treated rats compared to the Nx-Veh rats, further demonstrating activated myofibroblast, tissue remodeling and fibrosis, key features of EndoMT. Importantly, RKER-012 treatment suppressed these markers along with its attenuation of vascular remodeling (Figure 6F).

### RKER-012 suppresses perivascular inflammation and aberrant immune response in the lungs of a SuHx rat model of PAH

**Figure 7:**
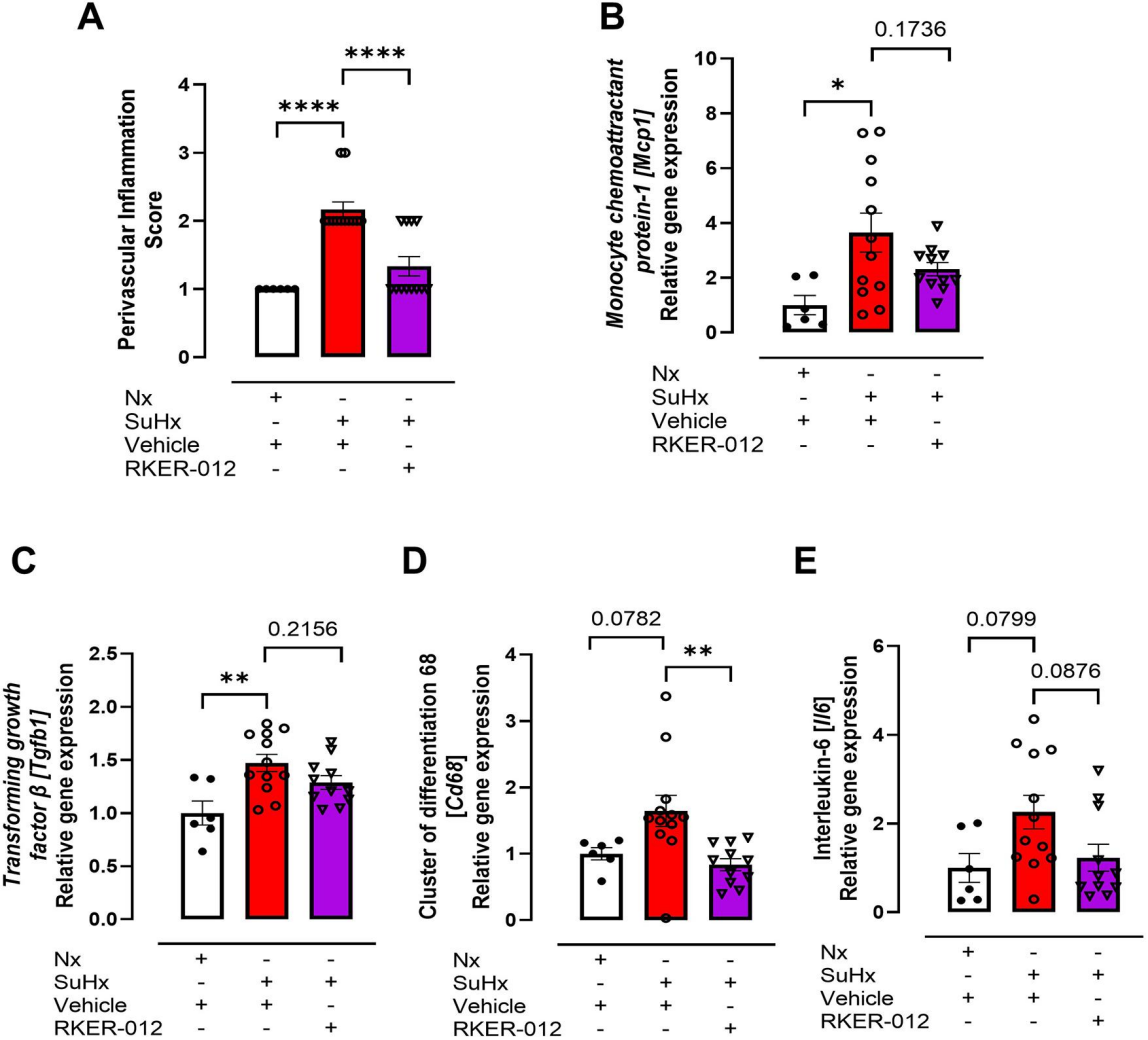
RKER-012 suppresses pulmonary inflammation and aberrant immune responses in an experimental preventative sugen hypoxia rat PAH model. **(A)** Perivascular inflammation score. Gene expression levels of **(B)** monocyte chemoattractant protein-1, *Mcp1*, **(C)** transforming growth factor β, *Tgfb1* **(D)** cluster of differentiation 68, *Cd68* and **(E)** interleukin-6, *Il6* and in lungs of normoxic rats treated with vehicle (Nx-Veh), Sugen hypoxia rats treated vehicle (SuHx-Veh) and Sugen hypoxia rats treated RKER-012 (SuHx-RKER-012). *n* = 6-12 in each group. Data are presented as Mean ± SEM. Analysis was performed by using one-way ANOVA and Tukey *post hoc* test (*p < 0.05, **p < 0.01, ****p < 0.0001).

Inflammation and aberrant immune cell modulation are integral to pulmonary vascular remodeling (4). Here, we evaluated the effect of RKER-012 on inflammation and aberrant immune response in the development of PAH in SuHx rats. Scoring of histopathological assessment at the perivascular regions of the SuHx-Veh rat lungs showed a significant increase in perivascular inflammation/fibrosis [*p* ≤ 0.0001] (Figure 7A) compared to normoxic controls (Nx-Veh). In contrast, SuHx rats treated with RKER-012 (SuHx-RKER-012) showed reduced perivascular inflammation [*p* ≤ 0.0001] compared to SuHx-Veh rats (Figure 7A). Due to differences in perivascular inflammation, animals were further assessed for inflammatory markers in whole lungs. SuHx-Veh rats had significantly increased monocyte chemoattractant protein-1, *Mcp1* [*p* = 0.0133] (Figure 7B) and transforming growth factor β, *Tgfb1* [*p* = 0.0030] (Figure 7C) expression compared to normoxic controls. An increasing trend in the expression of cluster of differentiation 68, *Cd68* [*p* = 0.0782] (Figure 7D) and interleukin-6, *Il6* [*p* = 0.0799] (Figure 7E) was observed in the lungs of SuHx-Veh rats compared to normoxic control. In contrast, SuHx rats treated with RKER-012 had significantly reduced expression of *Cd68* [*p* = 0.0058] (Figure 7D) compared to SuHx-Veh, while a decreasing trend in the expression of *Mcp1* [*p* = 0.1736] (Figure 7B), *Il6* [*p* = 0.0876] (Figure 7E), and *Tgfb1* [*p* = 0.2156] were observed (Figure 7C). Taken together, our results suggest that RKER-012 has anti-inflammatory properties.

### RKER-012 suppressed markers of cardiac injury, inflammation, and fibrosis in the right ventricle of SuHx rat model of PAH

**Figure 8:**
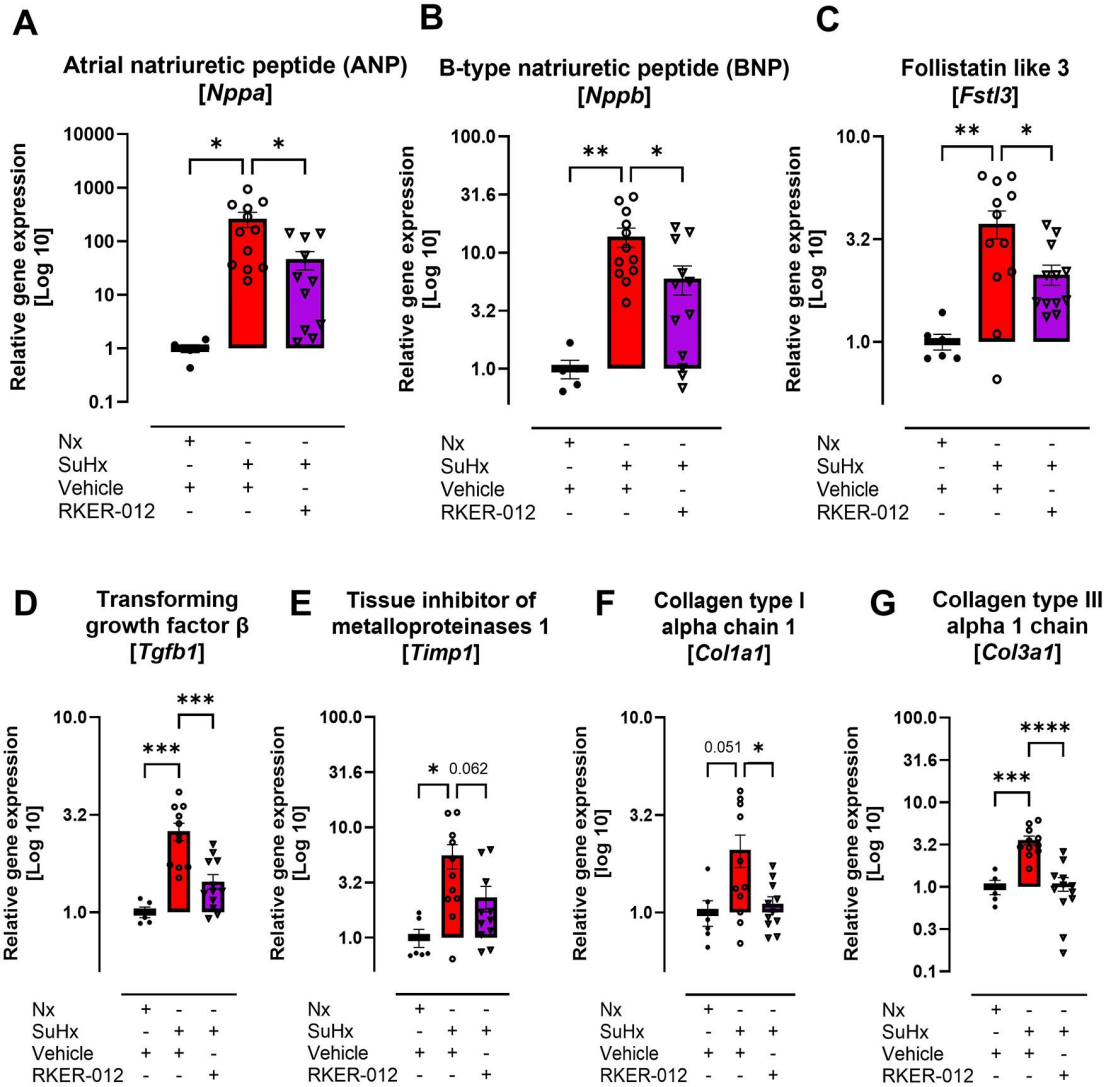
RKER-012 attenuated markers of cardiac hypertrophy and fibrosis in the right ventricle of rat sugen hypoxia rat PAH model. Gene expression levels **(A)** atrial natriuretic peptide (ANP), *Nppa,* **(B)** B-type natriuretic peptide (BNP), *Nppb,* **(C)** follistatin like 3, *Fstl3*, **(D)** transforming growth factor β, *Tgfb1*, **(E)** tissue inhibitor of metalloproteinases 1, *Timp1,* **(F)** collagen type I alpha chain 1, *Col1a1,* and **(G)** collagen type III alpha 1 chain, *Col3a1* in the RV of normoxia rats treated with vehicle (Nx-Veh), Sugen/hypoxia rats treated vehicle (SuHx-Veh), and Sugen/hypoxia rats treated RKER-012) (SuHx-RKER-012). *n* = 5-12 in each group. Data are presented as Mean ± SEM. Analysis was performed by using one-way ANOVA and Tukey *post hoc* test (*p < 0.05, **p < 0.01).

Right ventricular (RV) hypertrophy with increased afterload leads to cardiac injury, inflammation, and eventual fibrotic scarring. Therefore, we evaluated the effect of RKER-012 on the gene expression of markers of cardiac injury, inflammation and fibrosis in the RV of SuHx rats. In comparison to normoxic control (Nx-Veh), SuHx-Veh rats showed significant elevation of markers of cardiac injury, atrial natriuretic peptide (ANP), *Nppa* [*p* = 0.0426] (Figure 8A), B-type natriuretic peptide (BNP), *Nppb* [*p* = 0.0057] (Figure 8B), and follistatin like 3, *Fstl3* [*p* = 0.0016] (Figure 8C). In contrast, SuHx rats treated with RKER-012 showed a significant reduction in the marker of cardiac injury, (ANP), *Nppa* [*p* = 0.0317] (Figure 8A), (BNP), *Nppb* [*p* = 0.0310] (Figure 8B), and *Fstl3* [*p* = 0.0223] (Figure 8C) compared to SuHx-Veh. Similarly, in comparison to the normoxic control (Nx-Veh), RV of SuHx rats showed increase in expression of inflammatory markers, cluster of differentiation 68, *Cd68* (*p* < 0.01) (Supplementary Figure S4A), *c*luster of differentiation, *Cd8* (*p* = 0.53) (Supplementary Figure S4B), cluster of differentiation 11b, *Cd11b* (*p* < 0.05) (Supplementary Figure S4C). Importantly, RKER-012 treatment significantly decreased the expression of *Cd68* (*p* < 0.01) (Supplementary Figure S4A), *Cd8* (*p* < 0.01) (Supplementary Figure S4B), *Cd11b* (*p* < 0.05) (Supplementary Figure S4C) in SuHx-RKER-012 rats compared to SuHx-Veh rats. Likewise, in comparison to the normoxic control (Nx-Veh), RV of SuHx rats showed increase in expression of markers of fibrosis, transforming growth factor β*, Tgfb1* [*p* = 0.0002] (Figure 8D), Tissue inhibitor of metalloproteinases 1, *Timp1* [*p* = 0.0245] (Figure 8E), collagen type III alpha 1 chain, *Col3a1* [*p* = 0.0002] (Figure 8G), and collagen type I alpha chain 1, *Col1a1* [*p* = 0.0519] (Figure 8F). Treatment with RKER-012 significantly reduced expression of *Tgfb1* [*p* = 0.0005] (Figure 8D), *Col1a1* [*p* = 0.0347] (Figure 8F), and *Col3a1* [*p* ≤ 0.0001] (Figure 8G) relative to SuHx-Veh. Although statistically insignificant, the expression level of *Timp1* [*p* = 0.0619] (Figure 8D) showed a decreasing trend. In summary, together with the effects of RKER-012 observed in various aspects of pulmonary vascular remodeling and improved hemodynamics, our results also demonstrate that RKER-012 had a cardioprotective effect in SuHx rat model of PAH.

### RKER-012 has a direct cardioprotective effect in the RV of a pulmonary artery banding-induced RV injury

**Figure 9:**
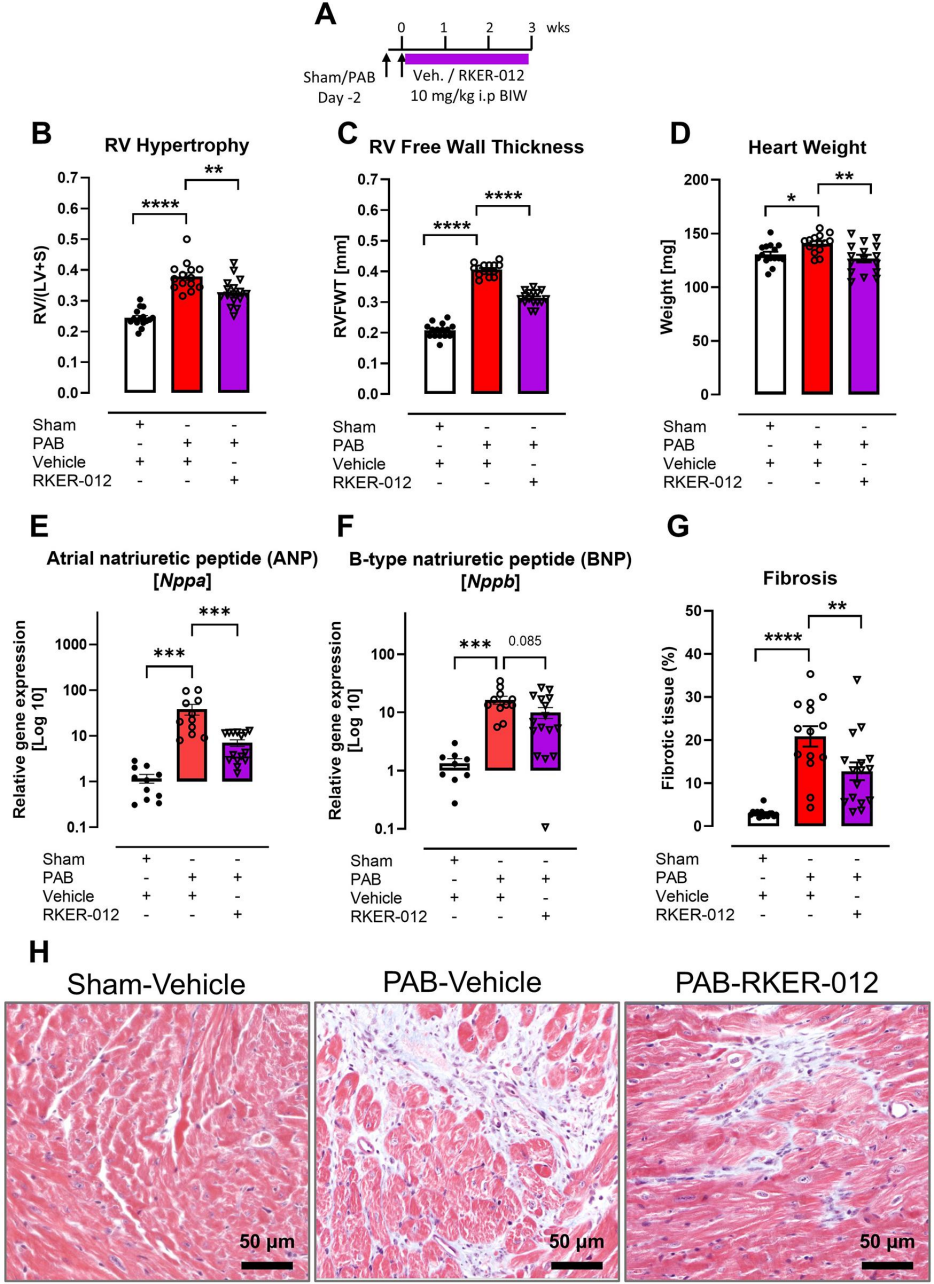
RKER-012 attenuates RV function in a pulmonary arterial banding mouse model. **(A)** Experimental approach to evaluate a direct cardioprotective effect of RKER-012 in a PAB model of pressure overload induced RV failure in male C57BL/6 mice. Suture ligature was placed in pulmonary artery for PAB or sham surgery was performed. PAB mice were treated with either vehicle or RKER-012 (10 mg/kg; i.p.) twice weekly for 3 weeks. **(B)** Right ventricular (RV) hypertrophy as assessed by Fulton Index, ratio of weight of Right Ventricle (RV) to weight of Left Ventricle (LV) and Septum (S) **(C)** Right ventricle free wall thickness (RVFWT), **(D)** Heart weight, **(E)** Gene expression levels of atrial natriuretic peptide (ANP), *Nppa*, and **(F)** B-type natriuretic peptide (BNP), *Nppb.* **(G)** Quantitative data showing percentage of fibrosis in the RV of Sham-Veh, PAB-Veh and PAB-RKER-012 groups. **(H)** Representative images of right ventricles stained with Masson trichrome. *n* = 14-15 in each group. Data are presented as Mean ± SEM. Analysis was performed by using one-way ANOVA and Tukey *post hoc* test (*p < 0.05, **p < 0.01, ***p < 0.001, ****p < 0.0001).

RV dysfunction and failure ultimately determine the mortality of PAH patients. Cardioprotective effects observed with RKER-012 in SuHx model are encouraging, but using SuHx model it is difficult to dissect out the treatment effects on RV independent of pulmonary vascular effects. Therefore, to assess the direct cardioprotective effects independent of pulmonary vascular effects, RKER-012 was evaluated in a mouse model of pulmonary arterial banding (PAB) (Figure 9A). PAB mouse is a model of compensated RV hypertrophy as a result of sustained elevated pulmonary artery pressure independent of pulmonary vascular pathology. To evaluate the sustained elevation of pulmonary artery pressure, a right heart catheterization was performed at the end of the study. In comparison to Sham-Veh mice, the systolic pulmonary artery pressure is elevated in both PAB-Veh and PAB-RKER-012 mice, demonstrating persistent increased afterload in the RV as a result of PAB (Supplementary Figure S5).

In comparison to Sham controls, PAB-Veh mice showed a significant increase in RV hypertrophy [+54.8%; *p* ≤ 0.0001] (Figure 9B), right ventricle free wall thickness [+95.8%; *p* ≤ 0.0001] (RVFWT, Figure 9C) and heart weight [+7.7%; *p* = 0.0310] (Figure 9D). Importantly, PAB mice treated with RKER-012 (PAB-RKER-012) showed significantly reduced RV hypertrophy [−13.5%; *p* = 0.034] (Figure 9B), RVFWT [−22.9%; *p* ≤ 0.0001] (Figure 9C), and heart weight [−9.9%; *p* = 0.0015] (Figure 9D) relative to PAB-Veh. Furthermore, in comparison to the Sham controls, PAB-Veh mice showed a significant increase in the gene expression markers of cardiac injury, atrial natriuretic peptide (ANP), Nppa (*p* < 0.001) (Figure 9E) and B-type natriuretic peptide (BNP), Nppb (*p* < 0.001) (Figure 9F). Similarly, in comparison to the Sham control, RV of PAB-Veh mice showed a significant increase in fibrosis (*p* < 0.0001) (Figures 9G,H). In contrast, PAB mice treated with RKER-012 (PAB-RKER-012) showed significantly reduced Nppa (*p* < 0.001) (Figure 9E) and fibrosis (*p* < 0.01) (Figures 9H,G) in comparison to PAB-Veh. Also, expression levels of Nppb (*p* = 0.085) showed a decreasing trend in PAB-RKER-012 rats compared to PAB-Veh (Figure 9F). Taken together, our results demonstrate that RKER-012 had a direct cardioprotective effect on RV independent of pulmonary vascular effects.

KER-012 altered serum proteins associated with inflammation and extracellular matrix remodeling pathways in healthy post-menopausal women.

**Figure 10:**
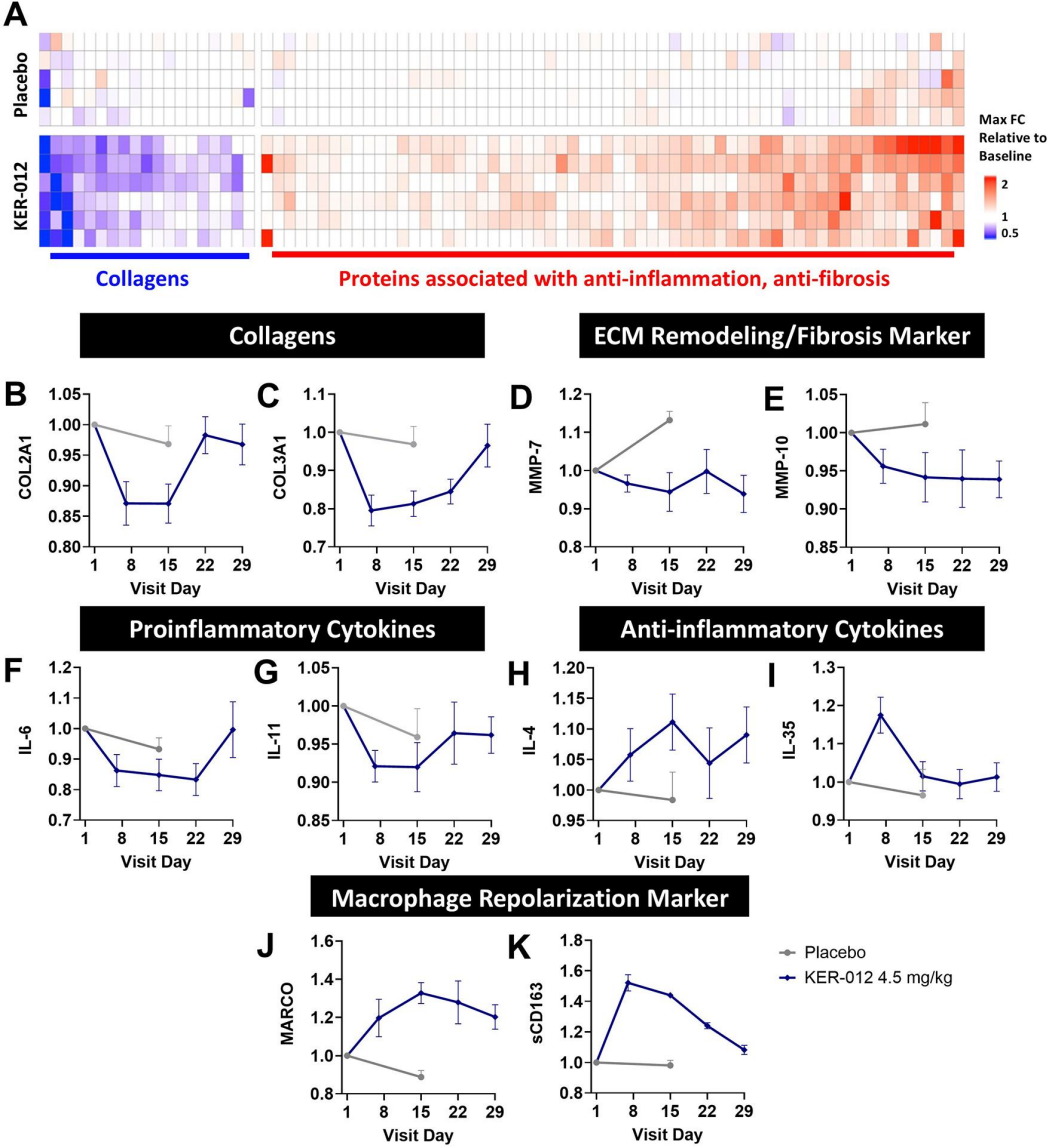
Administration of KER-012 altered serum proteins associated with inflammation and extracellular matrix remodeling pathways. **(A)** Heat map represents the maximal change per protein in each participant over 29 days post one dose of placebo or 4.5 mg/kg, KER-012. Representative examples of serum proteins altered by KER-012 administration in post-menopausal women. KER-012 decreased serum collagens **(B,C)**; extracellular matrix remodeling/fibrosis markers **(D,E)**; and proinflammatory cytokines **(F,G)**; increased anti-inflammatory cytokines **(H,I)** and markers of macrophage repolarization **(J,K)**. Data are presented as mean fold change from baseline ± SEM, *N* = 5-6 per group.

KER-012 was explored in a Phase 1 clinical trial in healthy post-menopausal women to evaluate safety and tolerability (54). While clinically healthy, this population is at greater risk for subclinical disease, including heart disease, which could potentially provide information on the mechanism of action and proof-of-biology of KER-012 in disease processes. Using the SomaScan platform, a comprehensive serum-wide proteomic analysis was performed to explore serum proteomics as a part of this study. Eighty-one proteins were significantly altered vs. placebo after KER-012 administration. Of these, 63 proteins were upregulated and 18 were downregulated vs. placebo. Figure 10A shows maximal change per protein in each participant over 29 days post one dose of placebo or 4.5 mg/kg KER-012, representing alteration of serum collagens, anti-inflammatory and anti-fibrotic proteins consistent with the predicted mechanism of action of KER-012. KER-012 administration in post-menopausal women showed decreased serum collagens as indicated by COL2A1 (Figure 10B) and COL3A1 (Figure 10C); extracellular matrix remodeling/fibrosis markers as indicated by MMP-7 (Figure 10D) and MMP-10 (Figure 10E); and proinflammatory cytokines as indicated by IL-6 (Figure 10F) and IL-11 (Figure 10G); increased anti-inflammatory cytokines as indicated by IL-4 (Figure 10H) and IL-35 (Figure 10I) and markers of macrophage repolarization as indicated by MARCO (Figure 10J) and sCD163 (Figure 10K). These findings support that KER-012 has the potential to alter structural remodeling pathways.

## Discussion

The pathophysiology of PAH is complex and involves multifactorial components (3, 4, 55). One of the key pathological events that leads to a progressive increase in pulmonary vascular resistance and increased RV load is pulmonary vascular remodeling, resulting in narrowing of the pulmonary arteries. The existing therapies are primarily vasodilators targeting prostacyclin, endothelin, or nitric oxide pathways, and do not adequately address reverse remodeling of the diseased pulmonary vasculature. Several reports have previously demonstrated that exaggerated activin-class ligands, including Activins A/B and GDFs 8/11, are key mediators of the pulmonary vascular remodeling, including aberrant inflammation and fibrosis in PAH (11–13, 56, 57). BMPs, on the other hand, are important regulators of vascular homeostasis, including vascular integrity (17). Activins/GDFs activate SMAD2/3 signaling while BMPs, including BMP9, activate SMAD1/5/9 (58). Approval of sotatercept, ActRIIA-Fc ligand trap, a first-in-class activin signaling inhibitor, for the treatment of PAH, has opened new avenues to explore the therapeutic potential of safely inhibiting activin signaling to alleviate PAH. Here, we describe a novel activin ligand trap, KER-012/RKER-012, a modified ActRIIB-Fc ligand trap with BMP-sparing properties, thereby minimizing the risk of reducing BMP signaling, an effect associated with vascular abnormalities. KER-012/RKER-012, while sparing BMPs, retains strong binding to Activins and GDFs, which are the key regulators of pulmonary vascular pathology in PAH. Our results in this study demonstrate that RKER-012 attenuated cardiopulmonary hemodynamics, pulmonary vascular remodeling, endothelial-to-mesenchymal transition, inflammation and aberrant immune responses, cardiac hypertrophy and fibrosis in a preclinical model of PAH. Furthermore, our results demonstrate that RKER-012 had a direct cardioprotective effect in a PAB model of pressure overload induced RV failure. Additionally, our results from an exploratory serum proteomics analysis from the Phase I study in healthy post-menopausal women to identify biomarkers of KER-012 activity showed proteomic changes that are consistent with anti-fibrotic and anti-inflammatory activity observed with RKER-012 and the translatability of pharmacological properties from rodent models to humans.

Sotatercept was initially developed for hematological indications because of its properties to induce erythrocytosis (38–40). It was originally evaluated in anemias of various etiologies, capitalizing on its robust effects of promoting erythrocyte maturation and increasing RBC parameters (59–63). In the results of the clinical trials, sotatercept showed rapid increases in erythrocytes, which have been cited to increase the risk of thromboembolic events and features of hyperviscosity syndrome in patients with PAH in its label (16). Because of this, sotatercept has dose-limiting safety risks, potentially limiting full target engagement (10, 39). Due to this effect, close monitoring of hemoglobin levels is warranted to adjust the dose and potential safety signals. Notably, sotatercept also binds members of the BMP ligand class in the TGF-β superfamily. BMPs are generally considered positive drivers in maintaining vascular health (17). Diminished BMP activity has been associated with the development of PAH, in addition to other diseases where the vasculature is compromised (17). In patients with PAH, BMP signaling is reduced, thereby increasing the risk of impaired vascular integrity. Further reduction in BMP signaling in PAH patients could disrupt vascular integrity and, hence, increase the risk of bleeding. Adverse effects such as telangiectasias and bleeding have been observed in patients treated with sotatercept (9, 16, 64). In contrast to sotatercept, an ActRIIA-Fc ligand trap, KER-012 consists of a modified human ActRIIB extracellular domain (ECD) that is fused to a human immunoglobulin G (IgG)1 fragment crystallizable (Fc) domain via a short linker sequence. RKER-012 is a research molecule for preclinical evaluation, which has a murine IgG2a Fc in place of the human IgG1 Fc domain found in KER-012. Unlike the ECD of wild type ActRIIB, which binds to BMPs in addition to activins and GDFs, the modified ActRIIB ECD of KER-012/RKER-012 is designed to spare BMP binding. Sequence alignment of activins, GDFs, and BMP ligands revealed notable differences in the amino acid residues at the receptor-ligand binding interface, as predicted by the known ActRIIB:BMP9 structure. These inherent variations in the TGF-β ligand sequences within the receptor:ligand binding interface may account for the differential binding affinities of ligands to both wild-type and modified ActRII-based ligand traps. This feature is important because KER-012/RKER-012 is designed to specifically trap activins and GDFs that have been shown to drive the pro-proliferative cellular phenotype in PAH (7, 11, 12), while sparing binding to BMPs, such as BMP9, which has been reported to play an essential role in maintaining vascular integrity (65, 66).

BMP9/10 are critical to postnatal development of superficial vascular plexus in the retina and branching of the blood vessels during this period (34). Complete inhibition of BMP9 and BMP10 results in disruption of the retinal vessel development, whereas if signaling of either ligand is spared, the vasculature develops normally. Our results demonstrate that RKER-012, a murine analog of cibotercept, in contrast to RAP-011, a murine analog of sotatercept, did not inhibit BMP/ALK1-dependent vascular outgrowth in the neonatal mouse model, thereby indicating no induction of disrupted retinal vascular development. The absence of observed perturbation of retinal blood vessels of newborn mice treated with RKER-012, due to its BMP-sparing properties, supports the potential for reduced bleeding risk. Furthermore, to understand if increased RBC and thereby development of hyperviscosity syndrome could potentiate a bleeding risk, as pointed out in the sotatercept label (16), a SuHx rat model was evaluated for EPO-induced erythrocytosis-mediated hyperviscosity and bleeding risks. SuHx rat model was chosen as it would closely represent patients with PAH with compromised BMP signaling, along with disrupted endothelial integrity (67). Our data provide evidence that Sugen-hypoxia rats were prone to bleeding when HVS was introduced by EPO-induced erythrocytosis, along with severe thrombocytopenic effects, and demonstrated reduced survival. These findings suggest that increased blood viscosity, partly due to erythrocytosis, along with accompanying severe thrombocytopenia, may potentially raise the risk of bleeding in patients with chronic diseases associated with systemic hypoxia, where vascular integrity may be compromised, such as PAH. Importantly, our results show that KER-012/RKER-012 did not increase RBCs in NHPs and rats. More importantly, in the Phase I healthy volunteer study, KER-012 also showed no clinically meaningful changes in hemoglobin or RBCs (68). Taken together, the BMP-sparing characteristics, alongside the lack of erythrocytosis observed in KER-012/RKER-012, not only differentiate KER-012 from sotatercept but also support the potential of KER-012 to reduce bleeding risk associated with vascular integrity and erythrocytosis-mediated hyperviscosity syndrome.

Pulmonary vascular remodeling is the primary pathological feature of PAH, contributing to increased vascular resistance and elevated afterload on the RV. Several complex biological events contribute to the remodeling of pulmonary vasculature, such as dysregulated cell growth, activation of EndoMT, remodeling of the ECM, inflammation, modulation of immune cells, and fibrosis (69, 70). These events collectively lead to the harmful tissue remodeling seen in pulmonary vasculopathy and cardiomyopathy. Notably, findings from several studies have indicated that exaggerated activin-class ligands, including Activins A/B and GDFs 8/11 play a key role in pulmonary vascular remodeling, including aberrant inflammation and fibrosis in PAH (11–13, 50, 51). More recently, Faizah et al. demonstrated that the Activin A-Endothelin-1 axis governs pulmonary vascular remodeling in PAH, where ET-1 was identified as a downstream effector of Activin A, supporting the argument that the inhibition of Activin A as a strategy to rescue pulmonary vasculopathy, including ET-1 mediated (71). Our results demonstrate that KER-012/RKER-012, a modified ActRIIB-Fc ligand trap, retains strong binding to Activins and GDFs with minimal impact on BMP signaling compared to ActRIIA-Fc, showed a beneficial effect on cardiopulmonary parameters in a Sugen-Hypoxia rat model of PAH. In addition to improvements in hemodynamic parameters, RKER-012 also demonstrated vascular remodeling in SuHx rat model. RKER-012 also attenuated the muscularization of non-muscular arteries, a key pathological feature of vascular remodeling (64–66), further supporting the notion that targeting activin signaling is beneficial for improving pulmonary vascular remodeling. RKER-012 protected against endothelial dysfunction and EndoMT, which are other key pathological determinants of pulmonary vasculopathy in PAH, further strengthening the notion that targeting activin signaling is favorable for improving pulmonary vascular remodeling. Clearly, endothelial cell experiments have demonstrated that EndoMT could be stimulated by activin, and that activin signaling inhibition could attenuate mesenchymal transition (71, 72). A recent report by Faizah et al, demonstrated that overexpression of Activin A in pulmonary arterial endothelial cells induces EndoMT with upregulation of SNAIL and SLUG, where inhibition of Activin signaling by follistatin was more effective in attenuating EndoMT in comparison to ET-1 signaling inhibition by bosentan, a dual endothelin receptor antagonist (71). In this study, we found that inhibiting Activin signaling with RKER-012 reduced elevated *Spp1* levels, an upstream regulator of Snail/Slug (73). These results suggest a reduction in EndoMT and further support the role of Activin A in endothelial pathobiology. Additionally, inhibiting Activin signaling may be an effective strategy for targeting these pathological processes. Similarly, inflammation and aberrant immune cell modulation have been closely associated with PAH pathologies, including pulmonary vascular remodeling (4). Targeting SMAD2/3 signaling has been shown to reverse pulmonary EndoMT due to acute inflammatory insult (74). Infiltration of immune cells and the release of inflammatory cytokines, driven by endothelial dysfunction, lead to perivascular inflammation, further exacerbating pulmonary vascular remodeling (4, 75). In this study, histological scoring demonstrated that RKER-012 suppressed perivascular inflammation in the lungs of SuHx rats. RKER-012 significantly reduced the gene expression of inflammatory markers such as *Cd68*, while in some cases a decreasing trend was observed, such as *Mcp1*, *Il6*, and *Tgfb1*. The decreasing trend in some of these markers could be attributed to the variability across the samples potentially arising from highly heterogeneous nature of lung tissue, unlike that of heart tissue, which is relatively more homogenous. This hypothesis can be further supported by the detection of a more pronounced reduction in *Tgfb1* expression in heart samples than in lung samples. Taken together, suppression of perivascular inflammation and aberrant immune response in the lungs of a SuHx rat model of PAH by a modified ActRIIB-Fc ligand trap further validates that targeting activin signaling can impact multiple components of the complex pathologies to alleviate PAH. Additionally, while limited by the exploratory nature and small sample size of the study, serum proteomics data from Phase 1 healthy post-menopausal volunteers showed a preliminary alignment with the anti-inflammatory and structural remodeling effects observed preclinically. These findings offer an initial indication of how pharmacodynamics translate from rodent models to humans; however, further validation in larger, disease-specific cohorts is necessary to confirm these observations.

Progressive vascular remodeling ultimately leads to RV failure and mortality. PAH patients with genetic variants in BMPR2 have been known to have more severely compromised RV function than non-carriers (76, 77). Impairment of BMPR2 signaling results in an imbalance between SMAD1/5/9 and SMAD2/3 signaling (7). Activin receptor signaling has been implicated previously as an important contributor to pathological cardiac remodeling in multiple types of heart failure (14, 78–83). Elevated levels of circulating activin A in patients support a role for this ligand in abnormal remodeling of the myocardium and heart failure (81, 84, 85). Elevation of activin A tilts the balance towards SMAD2/3. Using both *in vitro* and *in vivo* approaches, activin receptor ligand traps have been successfully used to inhibit SMAD2/3 signaling as a strategy to restore the balance between SMAD1/5/9 and SMAD2/3, consequently rescuing cardiopulmonary pathobiology in experimental pulmonary hypertension (7, 11–13, 86). In the present study, the modified ActRIIB-Fc ligand trap demonstrated a protective effect on the RV by suppressing markers of cardiac injury, inflammation and fibrosis in the SuHx rat model of PAH. More importantly, RKER-012, a modified ActRIIB-Fc ligand trap, had a direct cardioprotective effect in a PAB model of pressure overload induced RV failure. Clearly, in both the SuHx rat model and the PAB mouse model, RV is impaired as shown by a significant increase in the gene expression markers of cardiac injury, *Nppa* and *Nppb*. However, in PAB-mouse model, RKER-012 showed a significant reduction in the gene expression of *Nppa* while a decreasing trend was shown in the gene expression of *Nppb.* This difference in observation could potentially be due to differential regulation of gene expression of *Nppa and Nppb in* response to overactive activin signaling*.* MacDonell et al, reported that activin A overexpression increased *Nppa* gene expression by ∼1.8 fold while there were no significant changes observed with actvin A induced expression of *Nppb* (81). In this study also *Nppa* gene expression is substantially higher than *Nppb* in the RV of diseased heart. Furthermore, *Nppb* is increased in the failing heart. A 3-week PAB mouse model usually results in compensated RV hypertrophy, meaning the heart thickens to match the afterload without failing. While it increases fibrosis, it is considered a model of adaptive RV hypertrophy. In SuHx rat model, which is a preferred model for studying maladaptive RV hypertrophy and failure, a statistically significant decrease in both *Nppa* and *Nppb* was observed with RKER-012 treatment. Taken together, with the observations from the SuHx rat and the PAB mouse model, RKER-012 demonstrated cardioprotective effect.Overall, our results from preclinical models suggest that a modified ActRIIB-Fc ligand trap with BMP sparing properties and lack of erythropoietic effect has the potential to improve PAH by targeting multiple components in a complex pathobiology, such as dysregulated cell growth, activation of EndoMT, remodeling of the ECM, inflammation, modulation of immune cells, and fibrosis. However, due to unanticipated adverse events involving pericardial effusion, the TROPOS trial (NCT05975905), a randomized, double-blind, placebo-controlled, global Phase 2 clinical trial to evaluate cibotercept in combination with background therapy in patients with PAH, was early terminated. Nonetheless, the preclinical findings provide evidence that there is room for improvement in optimizing an activin receptor ligand trap to target overactive activin/GDF signaling in PAH while sparing BMP signaling and minimizing HHT-like phenomenon, vascular integrity, RBC effect, and simultaneously improving pulmonary vascular remodeling to alleviate PAH.

## Data Availability

The original contributions presented in the study are included in the article/Supplementary Material, further inquiries can be directed to the corresponding author.

## Supplementary material

Supplementary Figure S1 Negative Stain EM showed low resolution structure of KER-012 ligand trap in complex with human mature activin A. **(A)** Model of KER-012 ligand trap in complex with activin A generated by AlphaFold in ribbon (*left*) and surface (*right*) representation. **(B)** Representative negative EM 2D class averages of KER-012 [modified ActRIIB ECD (ActRIIB*)-Fc] with activin A obtained using cryoSPARC. The 2D classes show different orientations of the “ligand trap” in complex with activin A.

Supplementary Figure S2 Multiple Sequence Alignment of Relevant TGF-β Growth Factors. The figure depicts sequence homology across multiple TGF-β molecules.

Supplementary Figure S3 KER-012/ RKER-012 effect on RBC in non-human primates and SuHx rat model. Figures showing that there was no change in red blood cell number in **(A)** a non-human primate treated with 0 (Vehicle), 3, 10, and 50 mg/kg KER-012, s.c. Q2W and **(B)** SuHx rat treatment with 10 mg/kg RKER-012 s.c BIW. Data are presented as Mean ± SEM. Analysis was performed by paired Student t-tests at each dose. *N* = 6/sex/group.

Supplementary Figure S4 RKER-012 attenuated markers of inflammation in the right ventricle of SuHx rat PAH model. Gene expression of the inflammatory markers **(A)** cluster of differentiation 68, *Cd68,* **(B)** cluster of differentiation, *Cd8*, and **(C)** cluster of differentiation 11b, *CD11b*. Treatment with RKER-012 attenuated the increase for all three markers. Data are presented as Mean ± SEM. Analysis was performed by using one-way ANOVA and Tukey *post hoc* test (**p* < 0.05, ***p* < 0.01).

Supplementary Figure S5 PAB increased systolic pulmonary artery pressure (sPAP) in mice. Mice with pulmonary artery banding (PAB-Veh and PAB-RKER-012 mice) showed elevated sPAP in comparison to Sham-Veh mice. Mean ± SEM. Analysis was performed by using one-way ANOVA and Tukey *post hoc* test (****p* < 0.001, *****p* < 0.0001).
