# Supplementary material for: RKER-012, a modified ActRIIB-Fc ligand trap with BMP sparing properties, attenuates pathological features of experimental pulmonary arterial hypertension
Source: Front Cardiovasc Med. 2026 Jun 24;13:1827438. doi: 10.3389/fcvm.2026.1827438 (PMC13341533; doi:10.3389/fcvm.2026.1827438)
Supplement: Supplementary file 1 [file Table1.docx]

**Supplementary Table 1. Forward and reverse primer sequences for PAH-associated markers in rat and mouse.**

| **Gene** | **Species** | **Forward primer, 5’ – 3’** | **Reverse primer, 5’ – 3’** |
| --- | --- | --- | --- |
| ***Tbp*** | Rat | TTACGGCACAGGGCTTACTC | GGAAGAGTTGTGGGGTCTGG |
| ***B2m*** | Rat | GTGTCTCAGTTCCACCCACC | GACGGTTTTGGGCTCCTTCA |
| ***Acta-2* (αSMA)** | Rat | CATCCGACCTTGCTAACGGA | TGGCATGAGGCAGAGCATAG |
| ***Pai-1*** | Rat | ATCTTCAGCTCAACCCAGGC | GTTGGATTGTGCCGAACCAC |
| ***Sele* (E-Selectin)** | Rat | TTATCTGCACCACAGCGAGG | GAACACTGTACCCCTGCACA |
| ***Selp* (P-Selectin)** | Rat | CCCCTGGCAAGTGGAATGAT | CATAGAAGCCCGGGTAGCAG |
| ***Vcam-1*** | Rat | ACTGTGACCTGTCAGCGAAG | TTAGGGACCGTGCAGTTGAC |
| ***Ctgf*** | Rat | GCGCCTGTTCTAAGACCTGT | GGCTTGGCAATTTTAGGCGT |
| ***Spp1*** | Rat | CCAGCCAAGGACCAACTACA | CTGCCAAACTCAGCCACTTTC |
| ***Cd68*** | Rat | TCCAGGCTTCTCCACTGTTG | TTTGGGCTTGGAGCTGAACA |
| ***Il-6*** | Rat | CACTTCACAAGTCGGAGGCT | TCTGACAGTGCATCATCGCT |
| ***Tgfb1*** | Rat | AGGGCTACCATGCCAACTTC | CCACGTAGTAGACGATGGGC |
| ***Mcp1*** | Rat | CAGGTCTCTGTCACGCTTCT | GTAGTTCTCCAGCCGACTCA |
| ***Nppa* (ANP)** | Rat | CCTGGACTGGGGAAGTCAAC | ATCTATCGGAGGGGTCCCAG |
| ***Nppb* (BNP)** | Rat | CAGAAGCTGCTGGAGCTGATA | TCCGGTCTATCTTCTGCCCA |
| ***Fstl3*** | Rat | GGTGCTGAAGACACAGGTCA | TCCACTCCGTCGCAAGAATC |
| ***Timp1*** | Rat | GCCTCTGGCATCCTCTTGTT | AGCGTCGAATCCTTTGAGCA |
| ***Col1a1*** | Rat | GTACATCAGCCCAAACCCCA | GGGACTTCTTGAGGTTGCCA |
| ***Col3a1*** | Rat | TGCAATGTGGGACCTGGTTT | GGGCAGTCTAGTGGCTCATC |
| ***Cd8*** | Rat | GTGGAGGGAATGGGATTGGG | GGACATTTGCAAACACGCCT |
| ***Cd11b*** | Rat | CATGACCACCTCCTGCTTGT | GCTGCCCACAATGAGTGGTA |
| ***Rplp0 (36B4)*** | Mouse | AGATTCGGGATATGCTGTTGGC | TCGGGTCCTAGACCAGTGTTC |
| ***Nppa* (ANP)** | Mouse | AACCTGCTAGACCACCTGGA | ATCTATCGGAGGGGTCCCAG |
| ***Nppb* (BNP)** | Mouse | GAGTCCTTCGGTCTCAAGGC | AACAACTTCAGTGCGTTACAGC |
