# Supplementary figures and images for: RKER-012, a modified ActRIIB-Fc ligand trap with BMP sparing properties, attenuates pathological features of experimental pulmonary arterial hypertension
Source: Front Cardiovasc Med. 2026 Jun 24;13:1827438. doi: 10.3389/fcvm.2026.1827438 (PMC13341533; doi:10.3389/fcvm.2026.1827438)

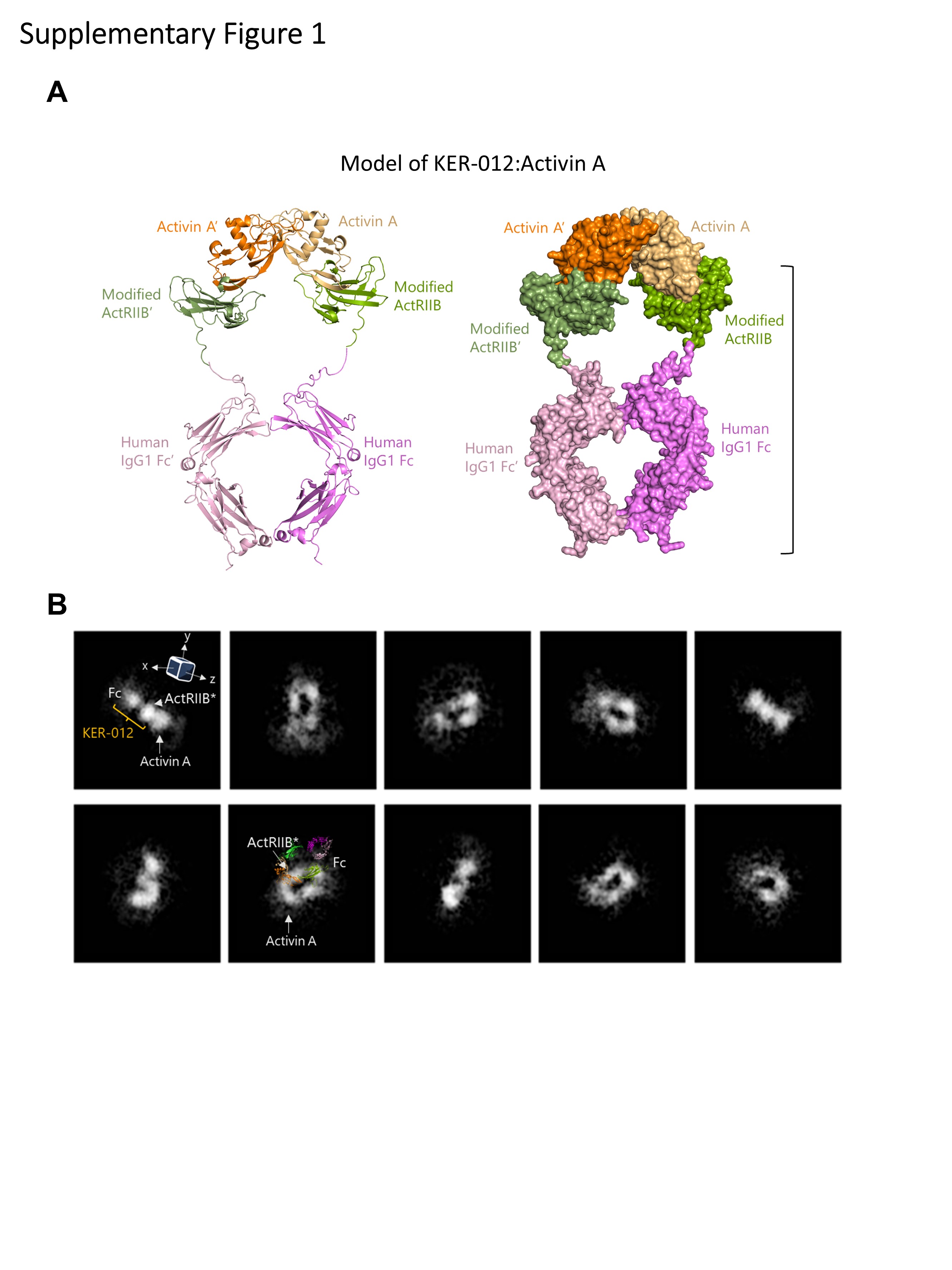

Supplement: Supplementary Figure S1 — Negative Stain EM showed low resolution structure of KER-012 ligand trap in complex with human mature activin A. (A) Model of KER-012 ligand trap in complex with activin A generated by AlphaFold in ribbon (left) and surface (right) representation. (B) Representative negative EM 2D class averages of KER-012 [modified ActRIIB ECD (ActRIIB*)-Fc] with activin A obtained using cryoSPARC. The 2D classes show different orientations of the “ligand trap” in complex with activin A. [file Image1.jpeg]

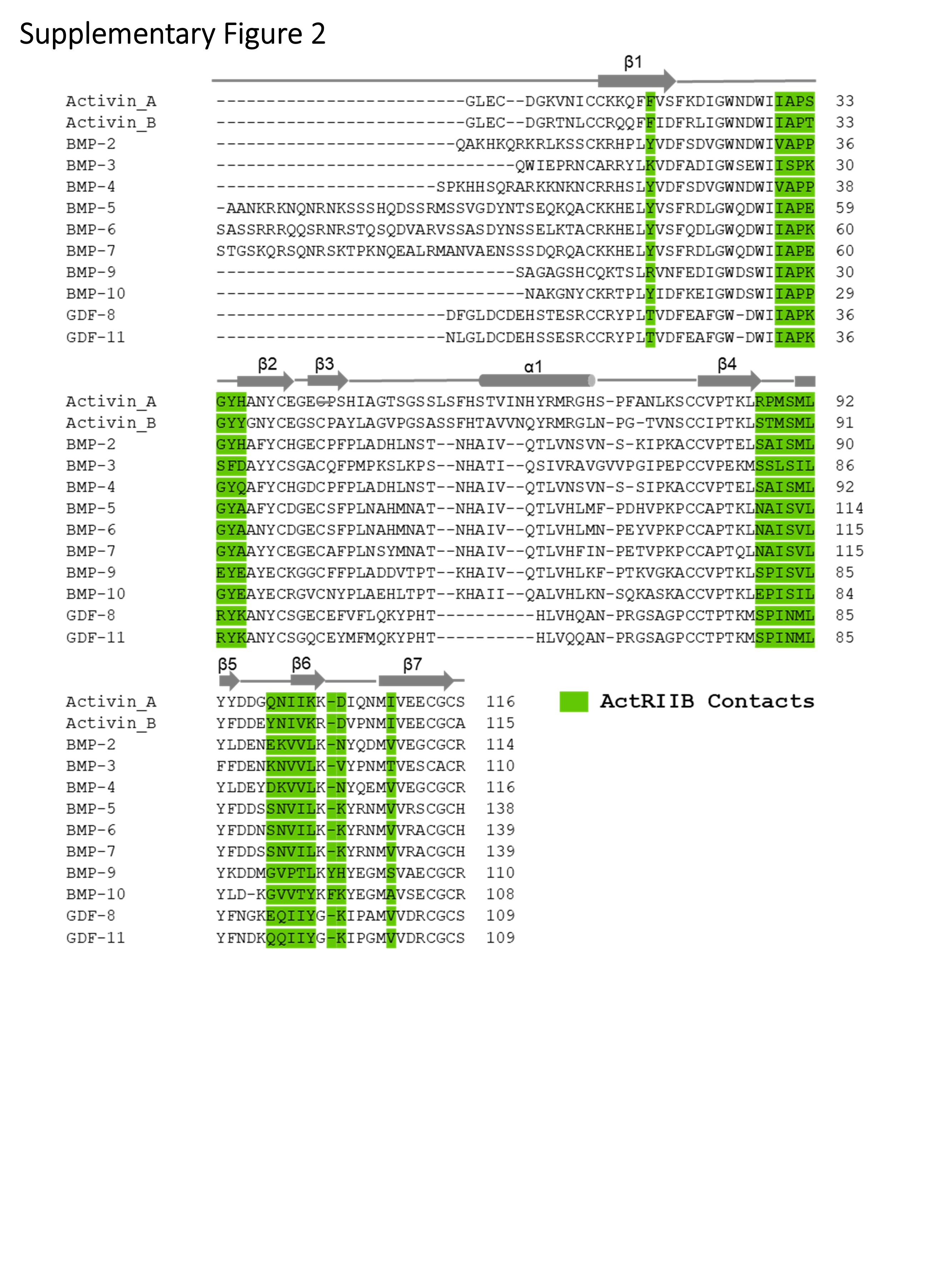

Supplement: Supplementary Figure S2 — Multiple Sequence Alignment of Relevant TGF-β Growth Factors. The figure depicts sequence homology across multiple TGF-β molecules. [file Image2.jpeg]

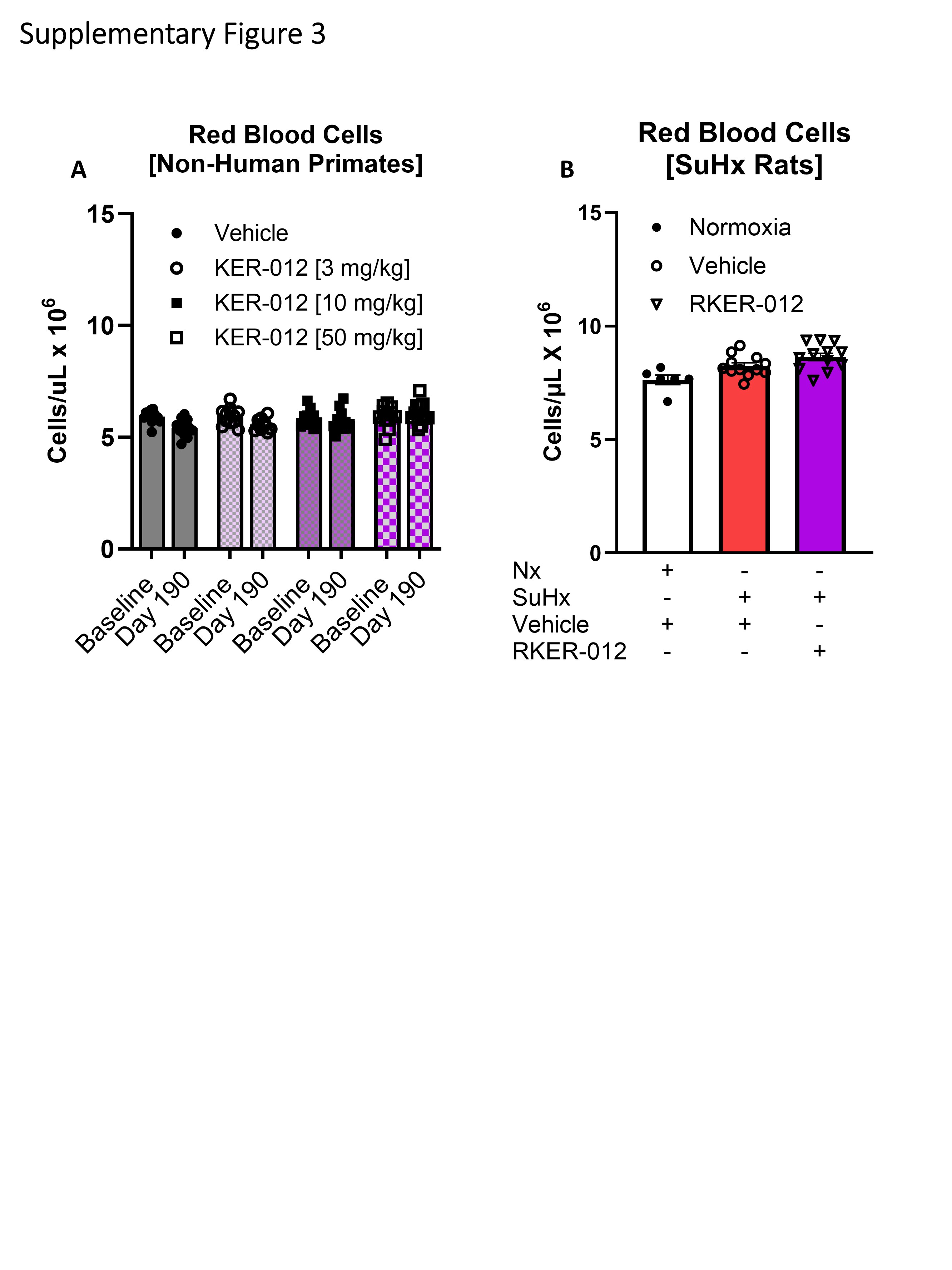

Supplement: Supplementary Figure S3 — KER-012/ RKER-012 effect on RBC in non-human primates and SuHx rat model. Figures showing that there was no change in red blood cell number in (A) a non-human primate treated with 0 (Vehicle), 3, 10, and 50 mg/kg KER-012, s.c. Q2W and (B) SuHx rat treatment with 10 mg/kg RKER-012 s.c BIW. Data are presented as Mean ± SEM. Analysis was performed by paired Student t-tests at each dose. N = 6/sex/group. [file Image3.jpeg]

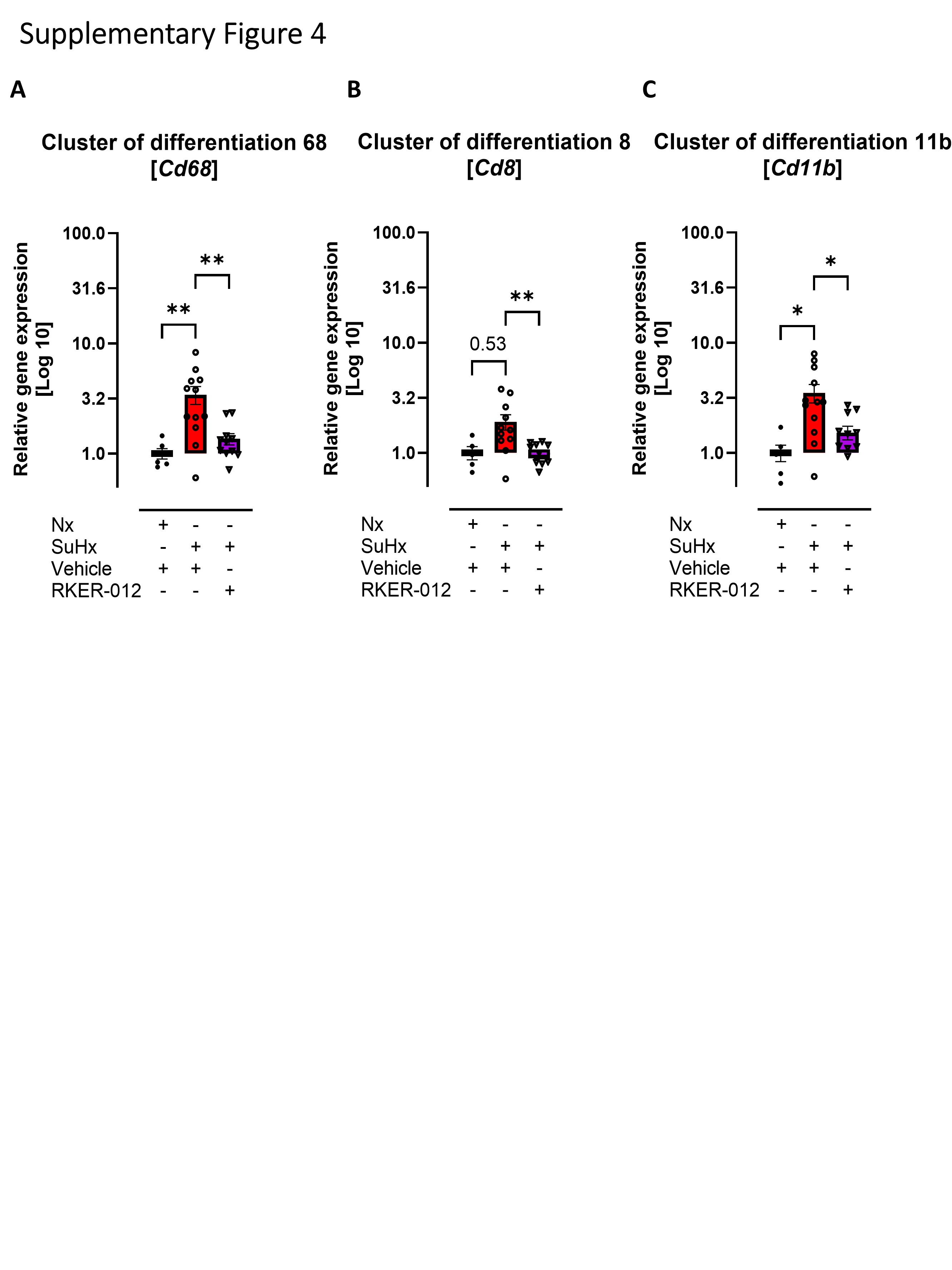

Supplement: Supplementary Figure S4 — RKER-012 attenuated markers of inflammation in the right ventricle of SuHx rat PAH model. Gene expression of the inflammatory markers (A) cluster of differentiation 68, Cd68, (B) cluster of differentiation, Cd8, and (C) cluster of differentiation 11b, CD11b. Treatment with RKER-012 attenuated the increase for all three markers. Data are presented as Mean ± SEM. Analysis was performed by using one-way ANOVA and Tukey post hoc test (*p < 0.05, **p < 0.01). [file Image4.jpeg]

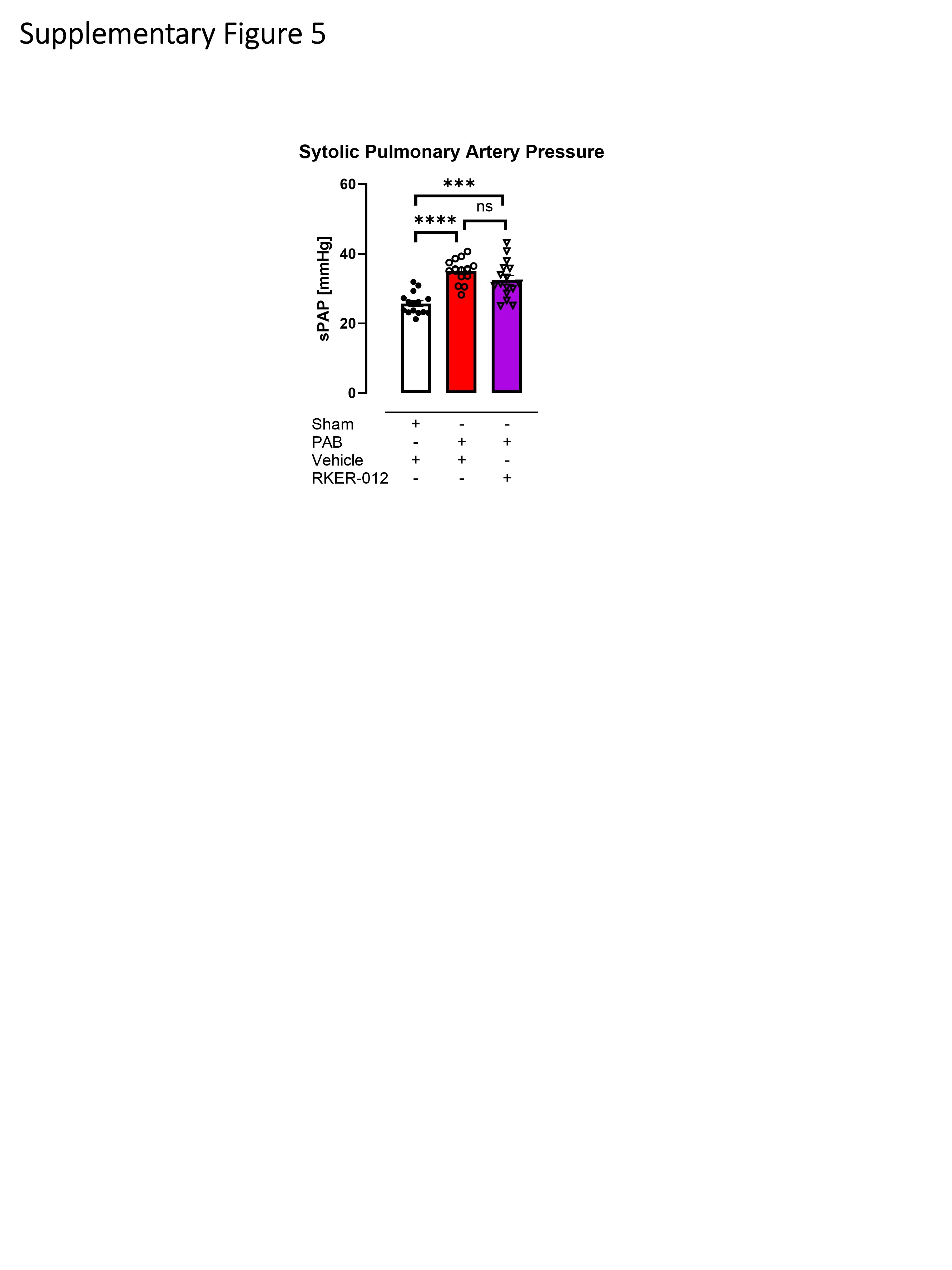

Supplement: Supplementary Figure S5 — PAB increased systolic pulmonary artery pressure (sPAP) in mice. Mice with pulmonary artery banding (PAB-Veh and PAB-RKER-012 mice) showed elevated sPAP in comparison to Sham-Veh mice. Mean ± SEM. Analysis was performed by using one-way ANOVA and Tukey post hoc test (***p < 0.001, ****p < 0.0001). [file Image5.jpeg]
